# Characterization of the inhalable fraction (< 10 μm) of soil from highly urbanized and industrial environments: magnetic measurements, bioaccessibility, Pb isotopes and health risk assessment

**DOI:** 10.1007/s10653-024-02009-z

**Published:** 2024-06-07

**Authors:** Stavroula Menegaki, Efstratios Kelepertzis, Zacharenia Kypritidou, Anastasia Lampropoulou, Vladislav Chrastný, Elina Aidona, Anna Bourliva, Michael Komárek

**Affiliations:** 1 Department of Geology and Geoenvironment, National and Kapodistrian University of Athens, 15784 Panepistimiopolis, ZographouAthens, Greece; 2 Department of Environmental Geosciences, Faculty of Environmental Sciences, Czech University of Life Sciences Prague, Kamýcká 129, 165 00, Prague-Suchdol, Czech Republic; 3 Department of Geophysics, Faculty of Geology, School of Geology, Aristotle University of Thessaloniki, Thessaloniki, Greece; 4 Directorate of Secondary Education of Western Thessaloniki, 56430 Thessaloniki, Greece

**Keywords:** Magnetite, Respiratory bioaccessibility, Source identification, Urban geochemistry

## Abstract

Soil in urban and industrial areas is one of the main sinks of pollutants. It is well known that there is a strong link between metal(loid)s bioaccessibility by inhalation pathway and human health. The critical size fraction is < 10 μm (inhalable fraction) since these particles can approach to the tracheobronchial region. Here, soil samples (< 10 μm) from a highly urbanized area and an industrialized city were characterized by combining magnetic measurements, bioaccessibility of metal(loids) and Pb isotope analyses. Thermomagnetic analysis indicated that the main magnetic mineral is impure magnetite. In vitro inhalation analysis showed that Cd, Mn, Pb and Zn were the elements with the highest bioaccessibility rates (%) for both settings. Anthropogenic sources that are responsible for Pb accumulation in < 10 μm fraction are traffic emissions for the highly urbanized environment, and Pb related to steel emissions and coal combustion in cement plant for the industrial setting. We did not establish differences in the Pb isotope composition between pseudo-total and bioaccessible Pb. The health risk assessment via the inhalation pathway showed limited non-carcinogenic risks for adults and children. The calculated risks based on pseudo-total and lung bioaccessible concentrations were identical for the two areas of contrasting anthropogenic pressures. Carcinogenic risks were under the threshold levels (CR < 10^–4^), with Ni being the dominant contributor to risk. This research contributes valuable insights into the lung bioaccessibility of metal(loids) in urban and industrial soils, incorporating advanced analytical techniques and health risk assessments for a comprehensive understanding.

**Supplementary Information:**

The online version contains supplementary material available at 10.1007/s10653-024-02009-z.

## Introduction

Metal(loid)s are common in all types of soils; their total concentration is derived from natural sources (volcanic eruption, dust storm, erosion, suspended soil fine particles) and numerous anthropogenic sources (vehicular exhaust emission, combustion, metal industries, mining/smelting) (Alloway, 2013; Luo et al., 2011; Wong et al., 2006). Soil is one of the main sinks of pollutants in every environment, collecting substantial levels of metal(loid)s over time (Huang et al., 2014; Luo et al., 2022; Tang et al., 2013). Soil contamination by metal(loid)s is a problem that all countries in the world are facing. Typically, urban, and industrial soils exhibit significantly higher mean concentrations of Cu, Hg, Ni, Pb, Sn and Zn relative to those in rural soils (Giordano et al., 2024; Hernández-Pellón et al., 2018). Generally, soils in urban areas are contaminated with Cd, Cu, Pb and Zn; soils at industrial sites can have distinct combinations of metal(loid) contaminants associated with different industries and their raw materials and products (Alloway, 2013; Raffa et al., 2021). Concentrations of elements in soils can be divided into ‘total’ and ‘available’. Total concentrations include all forms of the element in a soil while the ‘available’ concentration is an estimate of the fraction of that element which is present as either free ions, soluble complexes or readily desorbable (labile) forms (Alloway, 2013).

The presence of metal(loid)s in soils is associated with serious risks to human health (Hernández-Pellón et al., 2018; Li et al., 2020; Mukhtar & Limbeck, 2013; Wiseman, 2015). Metalloids can be transferred from contaminated soil to human body through three exposure pathways: ingestion, inhalation and dermal absorption (Guney et al., 2017; Li et al., 2021; Madrid et al., 2008). The human digestive or respiratory systems cannot absorb the total concentration of metal(loids) in the soil particles, therefore the term ‘bioaccessibility’ is used, which can be defined as the availability of a metal for absorption when dissolved in vitro in a body fluid (Guney et al., 2016). Generally, oral bioacccessibility has been recognized as the primary exposure path for human health (Billmann et al., 2023; Huang et al., 2014; Li et al., 2021). But inhalation bioaccessibility can lead to detrimental health consequences (Kastury et al., 2017; Tong et al., 2019), so inhalation bioaccessibility poses considerably greater risk to human health than oral (Kelepertzis et al., 2021).

Airborne particulate matter (PM) is a mixture of chemical species deriving either directly from sources (primary particles) of formed in the atmosphere through chemical reactions of gases and certain organic compounds. Airborne particulate matter (PM) in excess of 100 μm in diameter have a rather short atmospheric lifetime. Air quality guidelines and standards applicable to PM typically involve two categories, the PM_2.5_ fraction = aerodynamic diameters < 2.5 μm and PM_10_ fraction = aerodynamic diameters < 10 μm. In addition to PM, soil particles up to 100 μm can be inhaled via nose or mouth (Brown et al., 2013). It has been reported that only particles smaller than 10 μm can potentially deposit in the trachea-bronchial and alveolar region and pose a greater risk (Kastury et al., 2017). Furthermore, it is well established that the finer the particle size fraction, the higher the bioaccessibility, and the deeper the particles can penetrate into the human body (Kastury et al., 2017; Li et al., 2021; Madrid et al., 2008). In addition, soil particles < 10 μm can be suspended in the air for long periods of time from hours to days, and thus can be more easily inhaled by humans (Boim et al., 2021; Guney et al., 2017; Mukhtar & Limbeck, 2013). Thus, it is crucial to investigate the bioaccessibility of metalloids in < 10 μm soil size fraction through inhalation, as it represents a critical concern for human health. To simulate the conditions inside the lung and stomach in vivo experiments simulating fluids are used. The most common simulated lung fluids are the Artificial Lysosomal Fluid (ALF) with pH 4.5, which simulates the macrophage environment and the Gamble Solution (GS) with neutral pH 7.3 which simulates the alveoli environment (Guney et al., 2017).

Except for the chemical analysis, the last decades magnetic measurements have become a valuable tool for monitoring soil pollution. Ferromagnetic particles, usually iron oxides, are produced by a wide variety of technological processes at high temperatures (e.g. metallurgy, fossil fuel burning, cement production, etc.). The presence of ferromagnetic particles in urban areas is attributed to multiple sources such as: dust deposition from various industrial activities (e.g. metallurgy, etc.), road traffic, domestic heating and the presence of sludge materials (e.g. bricks, glass, tar, cement, etc.) or due to the presence of metallic waste (Liu et al., 2012; Verosub & Roberts, 1995). The observed relationship between anthropogenic pollution and magnetic signature of soils is extensively reported in the international literature, confirming the effectiveness of magnetic mapping of large urban areas in determining their degree of pollution (Hanesch & Scholger, 2005; Petrovsky et al., 2000; Wang et al., 2012). Additionally, several researchers have shown the correlation among magnetic properties and total elemental contents in soil, especially in urban agglomerations (Aidona et al., 2016; Bourliva et al., 2017; Xia et al., 2014), road dusts (Bourliva et al., 2016, 2018; Jordanova et al., 2014) or even indoor dust (Górka-Kostrubiec, 2015; Kelepertzis et al., 2019).

Lead (Pb) is a toxic metal that while occurring naturally in the environment, is predominantly introduced by human activities. The use of stable lead isotopes provides a valuable mean to trace the sources of soil pollution (Komárek et al., 2008; Reimann et al., 2012). Lead naturally occurs in four stable isotopes, including one non radiogenic isotope ^204^Pb (1%), and three radiogenic isotopes, ^208^Pb (52%), ^206^Pb (24%), and ^207^Pb (23%), which are the products of ^232^Th, ^235^U, and ^238^U decay chains, respectively (Ishida et al., 2023; Komárek et al., 2008; Wang et al., 2022). In earth sciences and especially in environmental sciences, the isotopic composition of Pb is typically expressed as ratios ^206^Pb/^204^Pb, ^206^Pb/^207^Pb, ^208^Pb/^206^Pb,with ^206^Pb/^207^Pb being the most preferred because it can be determined precisely analytically and is suitable for environmental applications (Komárek et al., 2008). Stable lead isotopes have been used in the earth sciences for over four decades with less application at environmental health investigations focused on humans (Gulson, 2008). Also, it is remarkable that fewer studies have tested the response of the Pb isotopic signal to different metal(loid) extraction methods, either single or sequential (Bacon & Hewitt, 2005; Ettler et al., 2006; Han et al., 2015; Kelepertzis et al., 2016; Teutsch et al., 2001). These studies have shed light on Pb distribution in different geochemical fractions with respect to Pb sources. However, studies that determined the isotopic composition of orally bioaccessible Pb are very limited (Farmer et al., 2011; Hiller et al., 2022; Li et al., 2015) whereas, to our knowledge, the isotopic composition of Pb extracted by a simulated lung extraction has been poorly investigated (Kelepertzis et al., 2021).

Numerus epidemiological studies have associated exposure to particles < 10 μm with severe respiratory and cardiovascular effects. Common annoyances are airway irritation, asthma exacerbation, inflammatory reactions, and fibrosis. More serious problems are cardiovascular disease, cerebrovascular disease, acute respiratory infections, and chronic obstructive pulmonary disease. Depending on the exposure type (short term or chronic exposure) and the characteristics of inhaled particles there are different effects. Also subjects with underlying diseases are facing greater risk (Oberdörster et al., 2005; Tong et al., 2019). A common approach to assess human health risk is the methodology developed by the United States Environmental Protection Agency (USEPA). By using USEPA equations the intake from the three exposure pathways can be evaluated considering the daily intake, time parameters and human characteristics (USEPA, 1989, 1997, 2002). USEPA models can examine carcinogenic risk and non-carcinogenic risks for adults and kids.

For this study, we selected two distinct areas: Athens, the capital city of Greece with population 3.2 million citizens was chosen as an example of a highly urbanized environment. Volos, located in Thessaly, Greece, was selected to represent an industrial setting. The selection of these two specific areas was based on the different sources of metal(loid) contamination with the aim to investigate the inhalable fraction in areas with contrasting anthropogenic pressures. In Greece, there is a limited number of recent bioaccessibility studies. Specifically, in these regions there is one research on oral bioaccessibility (< 100 μm) conducted in Athens (Kelepertzis & Argyraki, 2015) and another study on oral and lung bioaccessibility (< 100 μm) conducted in Volos (Kelepertzis et al., 2021). In a wider context, research on inhalation bioaccessibility has been relatively limited compared to the research on oral bioaccessibility, especially at European countries. This study is a combined methodology which aims to (i) to evaluate the magnetic signature of < 10 μm soil fraction from the two areas of investigation, (ii) evaluate the pseudo-total and inhalable bioaccessible content of metal(loid)s in the < 10 μm from a highly urbanized area (Athens) and an industrial area (Volos), (iii) determine the Pb isotope composition with the aim to trace the origin of Pb in < 10 μm soil fraction for both total and bioaccessible content, (iv) assess the non-carcinogenic and carcinogenic risks for adults and children in both settings. Although we recognize that PM sampling via filters would give important information in terms of human exposure to metal(loids) via inhalation in the specific areas, we focus here on the inhalable (< 10 μm) soil fraction with the aim to provide the scientific community with a holistic characterization of the soil particles that can be carried by the wind and reach the human respiratory system.

## Materials and Methods

### Study area and sample collection

Athens is one of the oldest cities in the world and has been the capital of modern Greece since 1834. The city lies within the expansive Athens Basin, an area spanning approximately 412 km^2^. The population growth of modern Athens started in 1920, establishing it as the largest and most densely populated urban center in the country for several years. Today, Athen’s basin in combination with Piraeus has a population of approximately 3,200,000 residents. Present-day Athens is characterized as a bustling urban metropolis with heavy traffic congestion. It encompasses an extensive residential network comprising apartment buildings and a network of roads linking Athens with other major urban and peri-urban areas. The city is also renowned for its wealth of archaeological and cultural attractions that draw tourists from around the world. In addition, natural green spaces, parks, and recreational areas provide a refreshing contrast to the urban landscape. Lastly, Athens functions as a financial hub, housing the headquarters of numerous companies and businesses.

Athens has a temperate Mediterranean climate with an average temperature of 18.3 °C. The city’s climate is characterized by a pattern of long hot and dry summers and mild, wet winters. The bedrock of Athens consists mainly of metamorphic rocks composed of marbles, schists and phyllites gneiss, as well as serpentinized blocks of varying dimensions. Clastic sedimentary rocks, limestone, dolomite, and Neogene and diluvial deposits are also present (Papanikoalou et al., 2004).

Volos is the capital of the Prefecture of Magnesia and belongs to the geographical district of Thessaly in central Greece. It is a coastal town situated along the bay of Pagasitikos Gulf. The area of the Municipality of Volos is 387.14 km^2^ and its permanent population is about 150,000 residents. The inception of the Industrial Zone in Volos is dating back in 1970, which brought about the establishment of many industries. During mid-1980s, Volos experienced a significant wave of deindustrialization. Today, the city includes a great number of industrial and commercial activities, as well as a harbor with a constant traffic of ferries and cruise ships. Notably, the region still houses some large industrial plants, such as steel and cement factories, several industries for agricultural and metallurgical products, plastics as well as food production factories.

The climate of Volos is of Mediterranean type with wet mild winters and hot dry summers with an average temperature of 16.8 °C. The bedrock of the wider area of Volos consists mainly of metamorphic rocks composed of gneiss, muscovite and mica-chlorite schists with marble intercalations, basic volcanic rocks and prasinite, clastic sedimentary rocks, limestone, and serpentinite occurrences. Also, it consists of sedimentary deposits with clay, sand and silt (Katsikatsos et al., 2018).

**Fig. 1:**
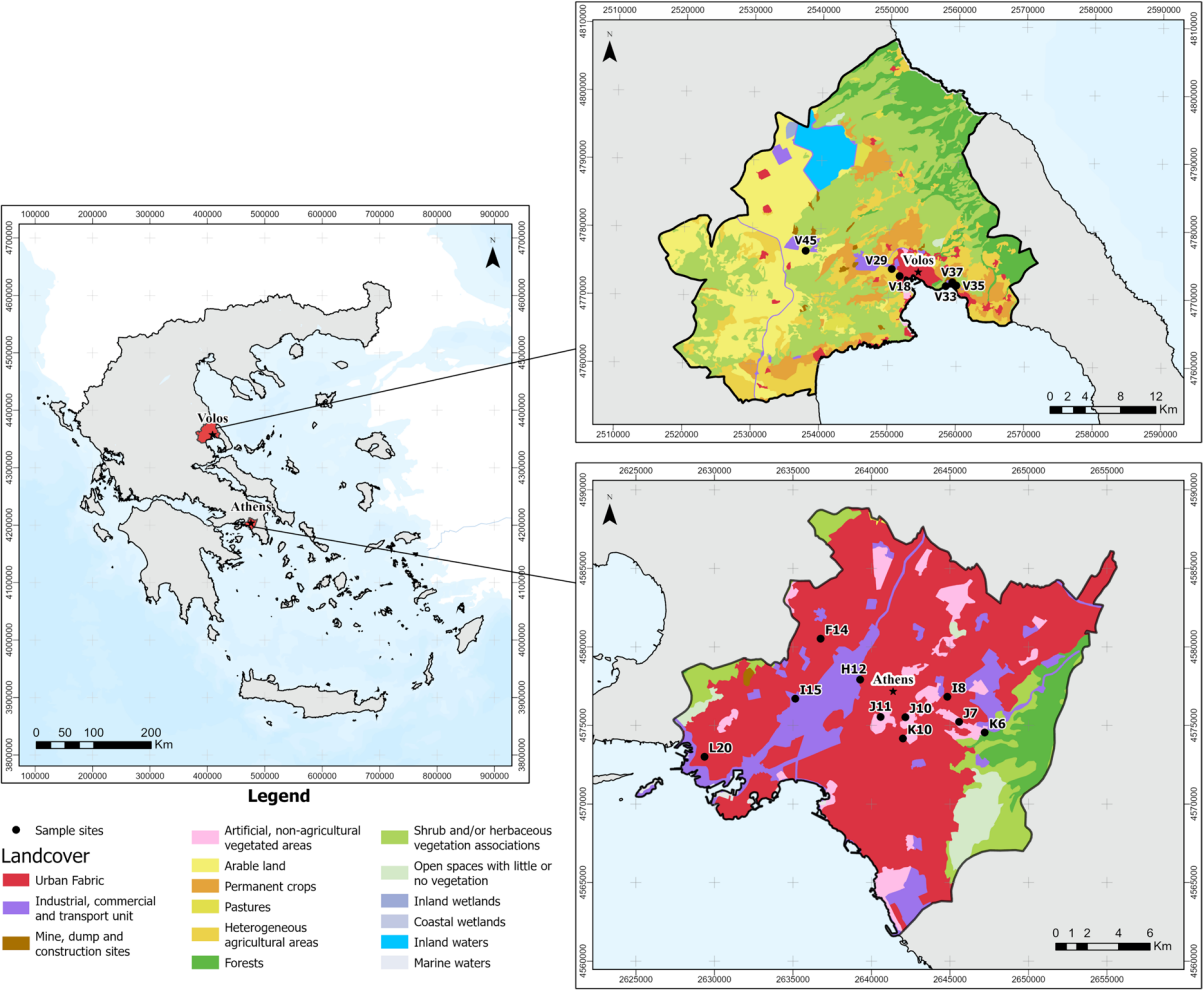
Landcover maps of the study areas showing the soil sampling locations

A total number of ten soil samples (0–10 cm depth) from Athens and six soil samples from Volos (0–10 cm depth) (Fig. 1) were selected from the sample database of previous investigations. The samples had been collected during spring of 2012 for Athens area and winter 2019 in the case of Volos. The criteria for sample selection were the high total content of trace metals of anthropogenic origin in < 100 μm soil fraction, focusing on Pb and Zn, as well the available quantity with the aim to extract sufficient sample for characterizing the inhalable fraction. Details on the sampling methodology are provided in the studies of Argyraki and Kelepertzis (2014) and Kelepertzis et al. (2020).

Mineralogical analysis of the ten selected samples from Athens revealed that the predominant minerals are calcite, quartz, muscovite and chlorite, whereas, quartz, calcite, albite, mica and chlorite were the major minerals present in Volos soil (Kelepertzis et al., 2020).

### Magnetic measurements

A combination of methodologies was employed to characterize and identify the magnetic minerals in soil samples with particle size less than 10 μm. The mass-specific magnetic susceptibility (*χ*) of the soil samples was assessed for low (0.46 kHz) frequency using a Bartingthon MS2 laboratory magnetic susceptibility meter, equipped with a dual frequency MS2B sensor at the Department of Geophysics, Aristotle University of Thessaloniki. The magnetic susceptibility value serves as an indicator of the concentration of strongly ferrimagnetic minerals, such as magnetite, within the sample.

Additionally, thermomagnetic analyses were conducted, involving the measurement of low-field magnetic susceptibility versus temperature (K–T curves). This analysis was obtained by continuously measuring from room temperature to 700 °C and back to room temperature using a Bartington furnace in free air. Thermomagnetic curves allow the determination of the Curie temperature and the stability of the magnetic carriers upon heating. All magnetic measurements were performed on soil particles with a size less than 10 μm.

### Extraction method for the < 10 μm soil fraction

The extraction of the < 10 μm particles was carried out through wet sieving and application of Stokes’ law, synthesizing appropriate methodologies from similar studies (Boim et al., 2021; Boisa et al., 2014; Ljung et al., 2008, 2011). Stokes law refers to the motion of a spherical body in a fluid and is defined as follows:

1 $$t = \frac{18\eta \cdot h}{{\left( {\rho s - \rho l} \right){\text{d}}^2 g}}$$

where,

η: viscosity = 0.89 mPa*s = 0.00089 kg/m*s.

h: the height from the suspension surface = 0.10 m.

ρs: the density of solids = 2,650 kg/m.^3^

ρl: the density of water = 1,000 kg/m.^3^

g: the acceleration of gravity = 9.81 m/s.^2^

d: the diameter of the particles = 10 μm.

**Fig. 2:**
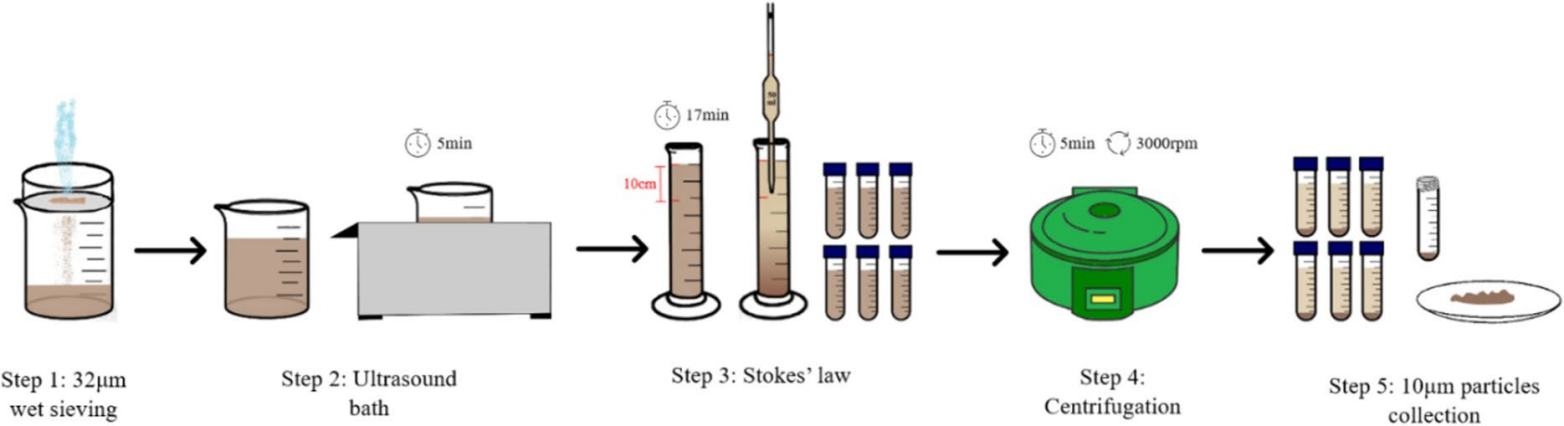
Extraction method for the separation of the < 10 μm fraction of soil samples

By using this formula, it was calculated that all particles smaller than 10 μm in diameter would have fallen 10 cm in the tube after about 17 min. Firstly, 40 g of < 100 μm soil fraction sample was subjected to wet sieving with < 32 μm membrane. The suspension was collected in a 1L beaker and was stirred and placed in an ultrasonic bath for 5 min. The suspension with < 32 μm fraction was transferred to a 1L volumetric cylinder. The cylinder was marked at 10 cm from the suspension level and left undisturbed for 17 min. At the end of this resting period, the first 10 cm of the suspension was carefully siphoned and transferred into a 1L beaker, and the process was repeated until the 10 cm space appeared nearly clear. After collecting all the < 10 μm suspension, the total volume was divided into 50 ml centrifuge tubes and centrifuged for 5 min at 3,000 rpm. Then, the supernatant solution was removed, and the remaining precipitate was placed in the oven at 60 °C. The detailed procedure described above can be visually represented in Fig. 2 for a clearer understanding. All laboratory glassware and the membrane (32 μm) were washed with a detergent, then soaked for 24 h in a 10% HNO_3_ acid solution and rinsed repeatedly with deionized water.

To assess the grain size of soil particles obtained by this method, two samples were examined using Scanning Electron Microscopy (SEM) (Jeol JSM 6360). The results indicated that almost all grains were within the desired size range. Details on the results of SEM are provided in Fig. S1 of the Supplementary material.

### Pseudo-total and lung bioaccessible content in the < 10 μm soil fraction

The pseudo-total concentration of metal(loids) in < 10 μm soil fraction was assessed using the USEPA 3050B protocol (1996). The pseudo-total concentrations of elements (As, Cd, Cr, Cu, Mn, Ni, Pb, and Zn) in digests were determined using ICP-OES and ICP-MS when necessary. To ensure quality control, reagent blanks, analytical duplicates, and standard reference materials for trace elements (SRM 2709a) were integrated into the analysis. Analytical duplicates exhibited relative percent differences of less than 20%. The recovery rates of SRM 2709a calculated as the measured pseudo-total contents compared to certified/reference and leachable values, ranged from 80 to 120% for most elements. Details regarding the quality control of chemical analyses are presented in Table S1 and S2 of the Supplementary Material.

To determine the bioaccessibility of metal(loids) through the respiratory tract, the conditions within the human lung was simulated in the laboratory. Between the two commonly applied simulated lung fluids (Gamble’s solution and Artificial Lysosomal Fluid, ALF), we applied in this study the ALF method. The main differences between Gamble’ solution and ALF are the lower pH and the higher organic content of ALF, resulting to higher extractable concentrations of elements in the ALF (Kastury et al., 2017; Ren et al., 2020). Our principal aim was to evaluate the maximum metal(loid) release of soil particles when in contact with a lung fluid.

The ALF method was applied to all soil samples. Specifically, 0.4 g of < 10 μm soil was weighed and placed in 50 ml centrifuge tubes. Subsequently, 40 ml of ALF solution was added to achieve a Liquid-to-Solid (L/S) ratio of 100 L/kg. The chemical composition of the ALF is shown in Table S3 in Supplementary Material. The sealed tubes were then placed on a rotating table at 37 °C, rotating at 250 rpm, for 24 h. After 24 h, the tubes were centrifuged for 15 min at 3,000 rpm. Following centrifugation, a portion of the supernatant was removed by using a disposable syringe and filtered through a 0.45 μm filter. Finally, one drop of concentrated HNO_3_ 65% was added to each tube and stored at 4 °C until further analysis. All laboratory glassware were washed with a detergent, then soaked for 24 h in a 10% HNO_3_ acid solution and rinsed repeatedly with deionized water. The lung simulated fluid was freshly prepared prior to the extraction of samples.

The bioaccessible concentrations of elements (As, Cd, Cr, Cu, Mn, Ni, Pb and Zn) were determined by ICP-OES and ICP-MS when necessary. For quality control, procedural blanks and analytical duplicates were added to each analytical batch. The results of procedural blanks analyses for the in vitro bioaccessibility experiments were predominantly lower than or near the instrument detection limits. The relative percent difference (RPD) was calculated for each pair of duplicates to assess samples homogeneity and methods precision, revealing RPD values lower than 20% for all elements. Details of the duplicate analyses are provided in Table S1 in Supplementary Material.

The percentage bioaccessible fraction (% bioaccessibility) was calculated as follows:

2 $${\text{\% bioaccessibility = (C}}_{{{\text{bio}}}} {\text{/C}}_{{{\text{total}}}} {\text{)*100}}$$

where C_bio_ is the inhalation bioaccessible concentration of metal(loid)s and C_total_ is the pseudo-total content of metal(loids).

### Lead isotopes

The Pb isotope analyses of pseudo-total and inhalable bioaccessible Pb in < 10 μm soil particles was conducted with an ICP-MS (iCap Q, Thermo Scientific, Germany). Correction for mass bias during the determination of the isotopic ratios was performed using analyses of SRM 981 (Common lead NIST, USA) after every two samples. The standard errors for measurement of the ^206^Pb/^207^Pb and ^208^Pb/^206^Pb were < 0.3% RSD and < 0.4% RSD, respectively. All soil samples were examined, and the results are presented as ratios of ^206^Pb/^207^Pb and ^208^Pb/^206^Pb. Lead isotope information were used to determine the origin of pseudo-total and bioaccessible Pb in the inhalable soil fraction.

### Health Risk assessment

The health risk assessment was performed for potentially exposed populations including adults and children. Pseudo-total and inhalable bioaccessible concentrations of metal(loid)s in < 10 μm fraction were both used for the calculations (Han et al., 2023; Ma et al., 2021). Bioaccessible portion is expected to make better estimation of the human health risk. The chronic daily intake for the respiratory track was calculated using the following equation:

3 $$CDI_{\left( {inh} \right)} = \frac{C \ast InhR \ast EF \ast ED}{{BW \ast AT \ast PEF}}$$

where CDI_(inh)_ (mg kg^−1^ day^−1^) was the daily intake via inhalation for each studied metal(loid); C (mg kg^−1^) was the concentration (pseudo-total or bioaccessible) of metal(loid); lnhR (m^3^ day^−1^) was the inhalation rate; EF (days year^−1^) was the exposure frequency; ED (year) was the exposure duration; BW (kg) was the body weight; AT (year) was the average time; PEF (m^3^ kg^−1^) was the soil-to-air particulate emission factor.

The health risk was assessed as non-carcinogenic and carcinogenic risk. In order to quantify the non-carcinogenic risk, the following equations were used:

4 $$HQ = \frac{CDI}{{RfD\dot{i}nh}}$$

5 $$HI = \sum^HQ$$

where HQ is the non-carcinogenic hazard quotient of each metal(loid); RfD_inh_ is the reference dose corresponding to the maximum inhaled dose required to avoid an adverse reaction when adsorbed. Hazard Index (HI) is the sum of HQs since the magnitude of adverse effects are cumulative for each element. When HI < 1 the non-carcinogenic risk is low, however the magnitude of risk increases as HI increases over safe level (= 1) (USEPA, 2002).

The carcinogenic risk (CR) which is the probability of an individual developing any type of cancer from lifetime exposure to carcinogenic hazards, was evaluated for As, Cd, Cr, Ni and Pb using equation:

6 $$CR = CDI \ast IUR$$

7 $$TCR = \sum^{\text{CR}}$$

where IUR (dimensionless) is the carcinogenic slope factor for inhalation track, and *TCR* is the total carcinogenic risk considering the specific metal(loids). A risk of < 10^−6^ can be regarded as negligible, 10^−6^ to 10^−4^ means tolerable risk, and > 10^−4^ means potentially high risk to humans (Ferreira-Baptista & De Miguel, 2005; USEPA, 2001). The parameter values used in calculations for the health risk assessment are given in Tables S4 and S5 in Supplementary Material.

### Data analysis

Minitab (v.17) Statistical Software was used for statistical analysis. Plotting of geochemical data and data obtained from the human health risk assessment model was performed with OriginPro 2016 (OriginLab Corp.).

## Results and discussion

### Magnetic parameters

Magnetic susceptibility (χ) values of the soil samples in < 10 μm fraction are presented in Table 1. Magnetic susceptibility (χ) ranged from 0.53 × 10^−6^ to 3.25 × 10^−6^ m^3^ kg^−1^ for the Athens samples exhibiting a moderate variability, and from 0.33 × 10^−6^ to 1.67 × 10^−6^ m^3^ kg^−1^ for the Volos samples. The results indicate the presence of ferrimagnetic phases. The magnetic susceptibility values showed no differences between the two areas (median 1.06 × 10^−6^ m^3^ kg^−1^ for both areas). The same samples but in another fraction (< 100 μm) have already been measured in previous research (Argyraki et al., 2018; Kelepertzis et al., 2021). Our new data compared to the published ones do not show any significant differences. This observation is previously reported in other studies (Bourliva et al., 2016).

**Table 1:** Statistical summary of magnetic susceptibility (χ), pseudo-total (EPA 3050B) and inhalable bioaccessible (ALF) concentrations of metal(loids) in < 10 μm soil fraction from the studied areas

χ (10^–6^ m^3^ kg^**−1**^**)**		Athens soil < 10 μm					Volos soil < 10 μm				
		Mean	Median	St. Dev	Minimum	Maximum	Mean	Median	St. Dev	Minimum	Maximum
		1.44	1.06	0.87	0.53	3.25	1.03	1.06	0.43	0.33	1.67
Pseudo-total	As	79	35	112	19	408	313	102	373	14	958
(mg kg^**−1**^**)**	Cd	0.90	0.61	0.93	0.33	3.62	0.56	0.54	0.12	0.4	0.74
	Cr	168	121	108	93	454	91	69	44	47	156
	Cu	117	105	54	40	224	77	38	74	16	223
	Mn	964	835	292	739	1540	725	645	347	351	1340
	Ni	240	134	265	106	1020	88	87	25	55	119
	Pb	424	382	328	129	1280	92	88	43	39	163
	Zn	422	341	265	143	1130	273	304	112	119	393
Lung bioaccessibility	As	11.5	9	8	4.36	34	89	46	98	6,7	270
(mg kg^**−1**^**)**	Cd	0.82	0.73	0.47	0.38	1.99	0.8	0.78	0.12	0.65	0.96
	Cr	14.4	13	3.94	9.82	21	21	17	8,1	14	36
	Cu	70	48	44	15	169	61	37	60	18	187
	Mn	818	777	223	513	1350	874	764	274	634	1410
	Ni	32	31	9.9	16	57	30	27	9,7	19	46
	Pb	341	218	311	104	1160	63	52	35	27	129
	Zn	217	208	123	45	440	244	282	120	67	402

Thermomagnetic curves indicate a drop of susceptibility at a range of 550 °C–560 °C, identifying an impure magnetite as the main magnetic carrier, since the Curie point is lower than 580 °C (Fig. S2 in Supplementary Material). On the heating curve we can observe a slight decrease of magnetic susceptibility from 200 °C–400 °C which can be related to the presence of maghemite (Jeleńska et al., 2004). In addition, the relatively smooth decrease in magnetic susceptibility between 450 °C and 550 °C, recorded in the samples may suggest the presence of a mixture of magnetite and maghemite or a wide grain-size distribution of magnetite (Magiera et al., 2011). The heating curves of both examined samples show a gradual increase of susceptibility value with a peak at 450 °C, demonstrating the Hopkinson effect. This peak has been frequently recorded in polluted soils (Jelenska et al., 2004, Bourliva et al., 2022). In all cases, the cooling curves are above the heating curves showing almost ten times enhanced susceptibility values after completion of the χ-Τ runs. This difference in the heating–cooling curves indicates the neo-formation of strongly magnetic phases during heating procedure.

### Pesudo-total and bioaccessible content in the < 10 μm soil fraction

The statistical summary of pseudo-total and lung bioaccessible concentrations in < 10 μm fraction of soil samples is presented in Table 1. The median pseudo-total concentrations of metalloids decreased in the order: Mn (835 mg kg^−1^) > Pb (382 mg kg^−1^) > Zn (341 mg kg^−1^) > Ni (134 mg kg^−1^) > Cr (121 mg kg^−1^) > Cu (105 mg kg^−1^) > As (35 mg kg^−1^) > Cd (0.61 mg kg^−1^) for Athens soil, and Mn (645 mg kg^−1^) > Zn (304 mg kg^−1^) > As (102 mg kg^−1^) > Pb (88 mg kg^−1^) > Ni (87 mg kg^−1^) > Cr (69 mg kg^−1^) > Cu (38 mg kg^−1^) > Cd (0.54 mg kg^−1^) for Volos soil. The pseudo-total concentrations of metal(loid)s (e.g. As, Mn, Pb and Zn) in most samples exhibited considerable variation reflected by their high relative standard deviation (Table 1), demonstrating the inherent heterogeneity of the particles or anthropogenic influences (Karim et al., 2013; Ungureanu et al., 2017). A general trend was observed indicating higher pseudo-total metalloid concentrations in the soils of Athens compared to those of Volos. The difference was especially clear for Cr, Cu, Mn, Ni and Pb, attributed to the presence of ophiolites in Athens that are responsible for Cr, Mn and Ni enrichment, and to traffic-related emissions in the case of Cu and Pb (Argyraki & Kelepertzis, 2014). Arsenic displayed a contrasting behavior, demonstrating higher concentrations in Volos soils as opposed to Athens attributed to adjacent mineralization that is rich in realgar and pyrite (Kelepertzis et al., 2020).

**Fig. 3:**
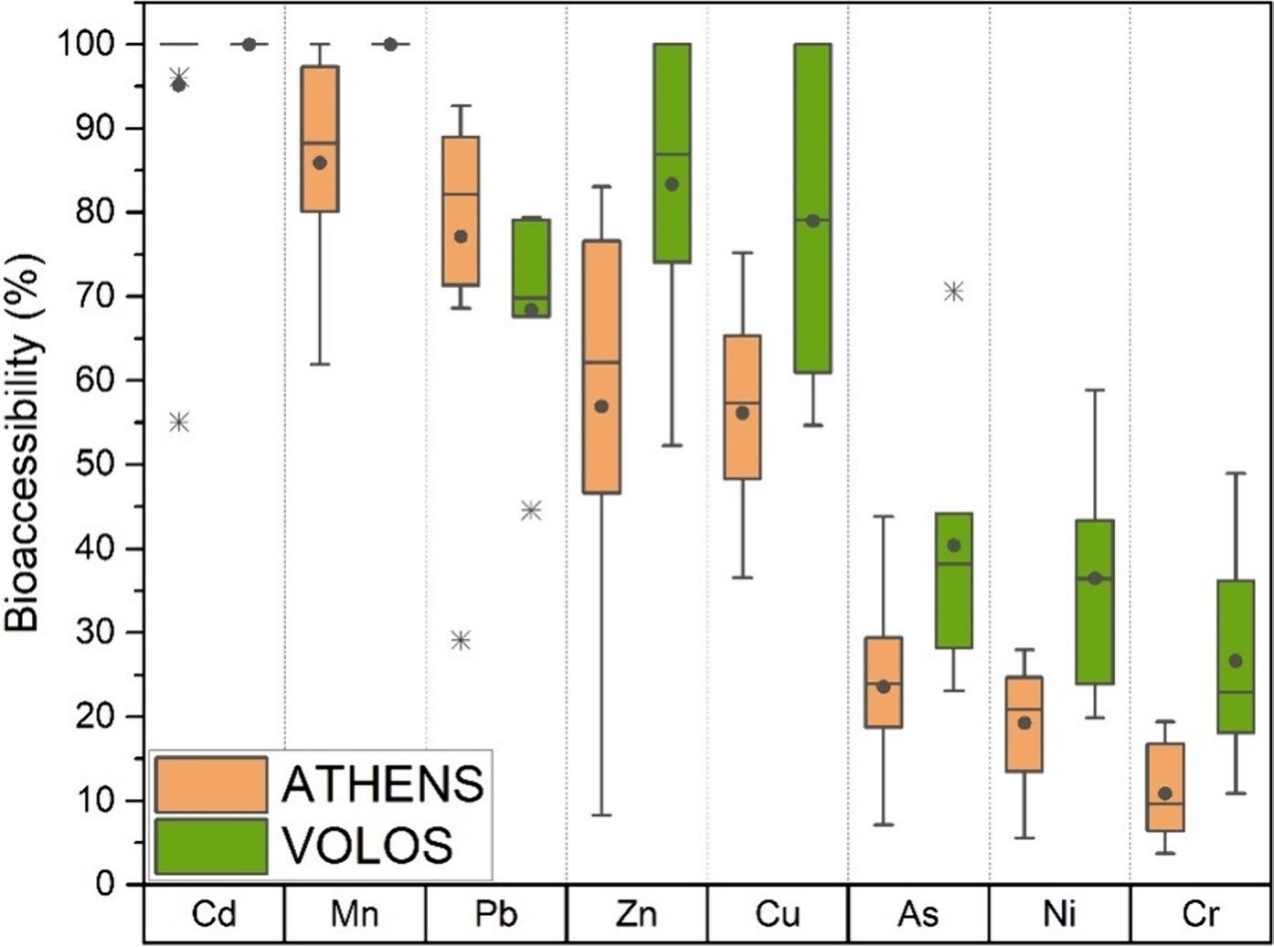
Lung bioaccessible fractions (%) of metal(loid)s in the < 10 μm soil fraction

The lung bioaccessibility of metalloids was calculated as the ratio of the bioaccessible concentration to the respective pseudo-total content (Fig. 3). The median bioaccessibility ratios decreased in the order: Cd (100%) > Mn (88%) > Pb (82%) > Zn (62%) > Cu (57%) > As (24%) > Ni (21%) > Cr (10%) for Athens soil, and Cd (100%) > Mn (100%) > Zn (86%) > Cu (74%) > Pb (69%) > As (39%) > Ni (34%) > Cr (25%) for Volos soil. In general, the bioaccessible percentage was found to be higher in Volos soils, with the exception of Pb which demonstrated slightly higher bioaccessibility percentages in Athens. Cadmium displayed a 100% bioaccessible rate in both Athens and Volos soils. Similar percentages of inhalation bioaccessibility for this element have been measured in soil samples from Brazil (Boim et al., 2021) and PM_10_ from Vienna, Austria (Falta et al., 2008). The high bioaccessibility percentage for both areas for most of metal(loids) can be attributed to the aggressiveness of the ALF solution because of its low pH (4.5) (Boim et al., 2021; Li et al., 2020; Wiseman & Zereini, 2014). In addition, the high fraction of metal(loids) released by the ALF solution is governed by the complexation capacity of some components present in ALF, resulting to ligand-induced elemental release (Hernández-Pellón et al., 2018).

Upon comparing the pseudo-total measurements in < 10 μm with the pseudo-total data in < 100 μm from previous researches (Kelepertzis & Argyraki, 2015; Kelepertzis et al., 2020), distinct patterns are emerged in the metalloid distribution for the soils of the two studied areas (Fig. S3 in Supplementary Material). In Athens soils, all the metalloids exhibited higher concentrations in the smaller particle size of < 10 μm. This was consistent with numerous studies highlighting that metal concentrations increase with decreasing particle size (Cao et al., 2012; Kong et al., 2011) which can be explained by the larger active surface of the fine particles. The fine particles are typically associated with greater proportion of Fe and Mn oxides and clay minerals that scavenge metal(loids) through adsorption processes (Luo et al., 2011). Moreover, the small size fraction of metal(loid) embedded particles of anthropogenic origin is also responsible for the preferential accumulation of metal(loids) in the < 10 μm fraction. Contrarily, metal(loids) concentrations in < 10 μm fraction are similar to those measured in < 100 μm for Volos soil (Fig. S3 in Supplementary Material). This pattern could be explained by the size of metal-bearing particles emitted by steel and cement industries occurring in Volos, being both small and large in size (Kelepertzis et al., 2021). More importantly, a comparison between lung bioaccessibility in < 10 μm and < 100 μm fractions for Volos soil shows that all elements exhibit higher concentrations and percentages (%) in the < 10 μm fraction (Fig. S4 in Supplementary Material). This is indicative of the preferential dissolution of particles < 10 μm in the lung solution compared to particles with larger size, demonstrating the occurrence of more bioaccessible forms of metal(loids) in the finest fraction.

Finally, a comparison of the present data for both pseudo-total and lung bioaccessible concentrations with data from other studies is shown in Tables S6 and S7 in Supplementary Material. Overall, soil samples in Athens show similar pseudo-total concentrations with soil from the other areas. On the contrary, soil in Volos exhibit either similar or lower concentrations, with the exception of the elevated levels of As. Compared to the European soil mean values (Foregs, 2005) and the worldwide soils (Kabata Pendias, 2011), soil in both study areas indicate signs of contamination. In terms of lung bioaccessible concentrations, concentrations in soil fraction < 10 μm from Athens show higher concentrations for almost all elements. A striking feature is the elevated lung bioaccessible concentrations of As and Mn in Volos samples.

#### Pb isotope analyses of pseudo-total and bioaccessible Pb in < 10 μm soil fraction

**Fig. 4:**
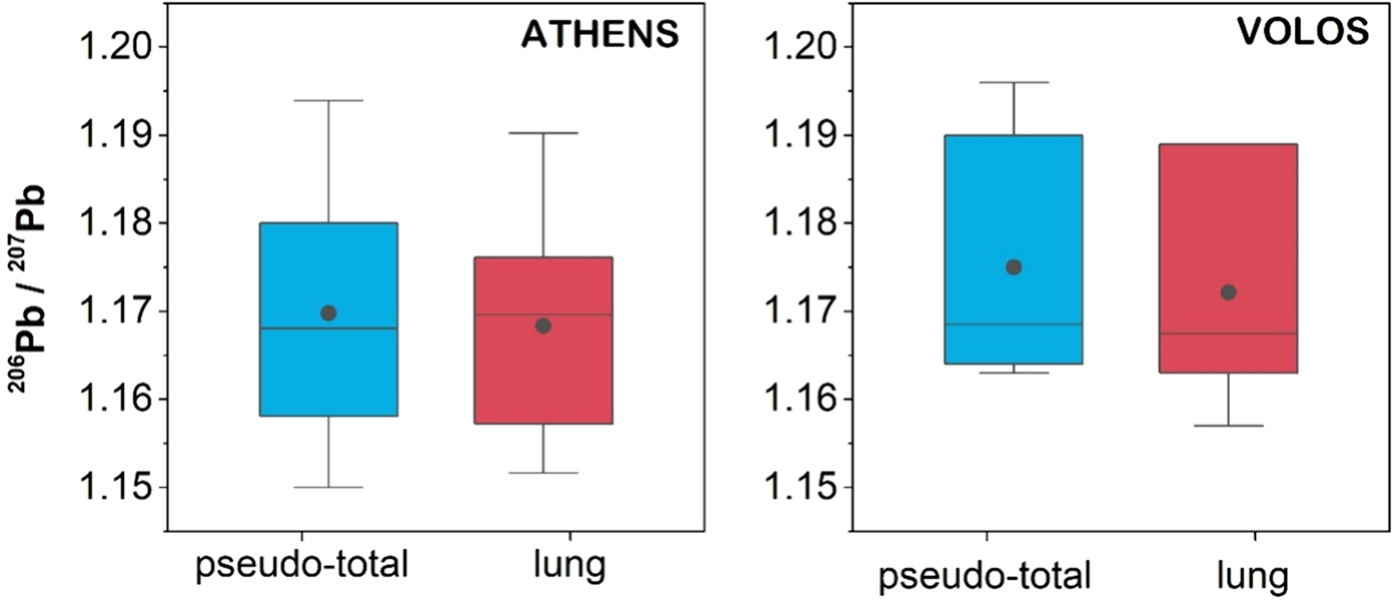
Variations in ^206^Pb/^207^Pb isotope ratios of the pseudo-total and lung bioaccessible Pb in < 10 μm soil fraction from the two studied areas

**Fig. 5:**
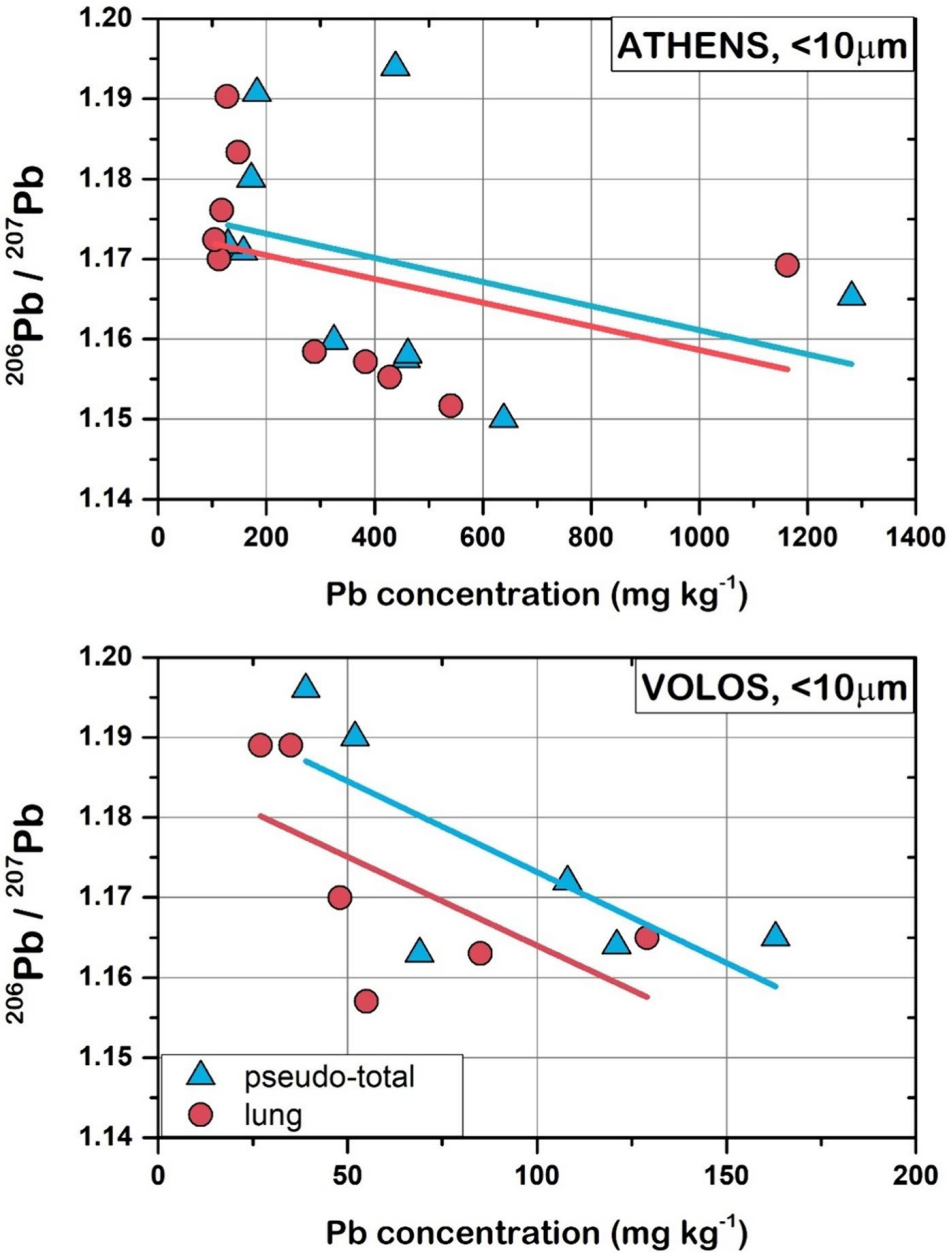
Relationships between Pb concentrations and ^206^Pb/^207^Pb ratios measured in < 10 μm soil fraction from the two studied areas

The results of Pb isotope compositions in the < 10 μm size fraction of soil samples from highly urbanized and industrial sites are presented in Table S8 in Supplementary Material. The ^206^Pb/^207^Pb ranged from 1.150 to 1.194 in pseudo-total digests and from 1.152 to 1.190 for lung bioaccessible Pb in Athens soil. With regard to Volos soil, the ^206^Pb/^207^Pb ratio of pseudo-total Pb ranged from 1.163 to 1.196, and from 1.157 to 1.189 for bioaccessible Pb. Generally, geological Pb exhibits higher ^206^Pb/^207^Pb and lower ^208^Pb/^206^Pb ratios and is less soluble in acidic solutions than Pb derived from anthropogenic sources (Komárek et al., 2008). With slight variations, the Pb isotope ratios measured for the lung bioaccessible fraction of Pb in the investigated soils were mostly consistent with the Pb isotope ratios of pseudo-total Pb in < 10 μm fraction for both areas (Fig. 4). This isotopic similarity is in agreement with findings by Wang et al. (2022) and indicates the similar Pb leachability in both extracts used to measure pseudo-total and lung bioaccessible Pb content. Such observations further highlight that the anthropogenic Pb fingerprint is well delineated in both implemented analytical protocols, suggesting the use of isotopic composition of bioaccessible Pb for tracing human exposure to soil Pb via the inhalation pathway. In addition, we observed a trend of increasing Pb concentrations (both pseudo-total and lung bioaccessible) with decreasing ^206^Pb/^207^Pb isotope ratios (Fig. 5), indicative of the higher contribution of anthropogenic Pb as Pb content in the < 10 μm soil fraction increases.

**Fig. 6:**
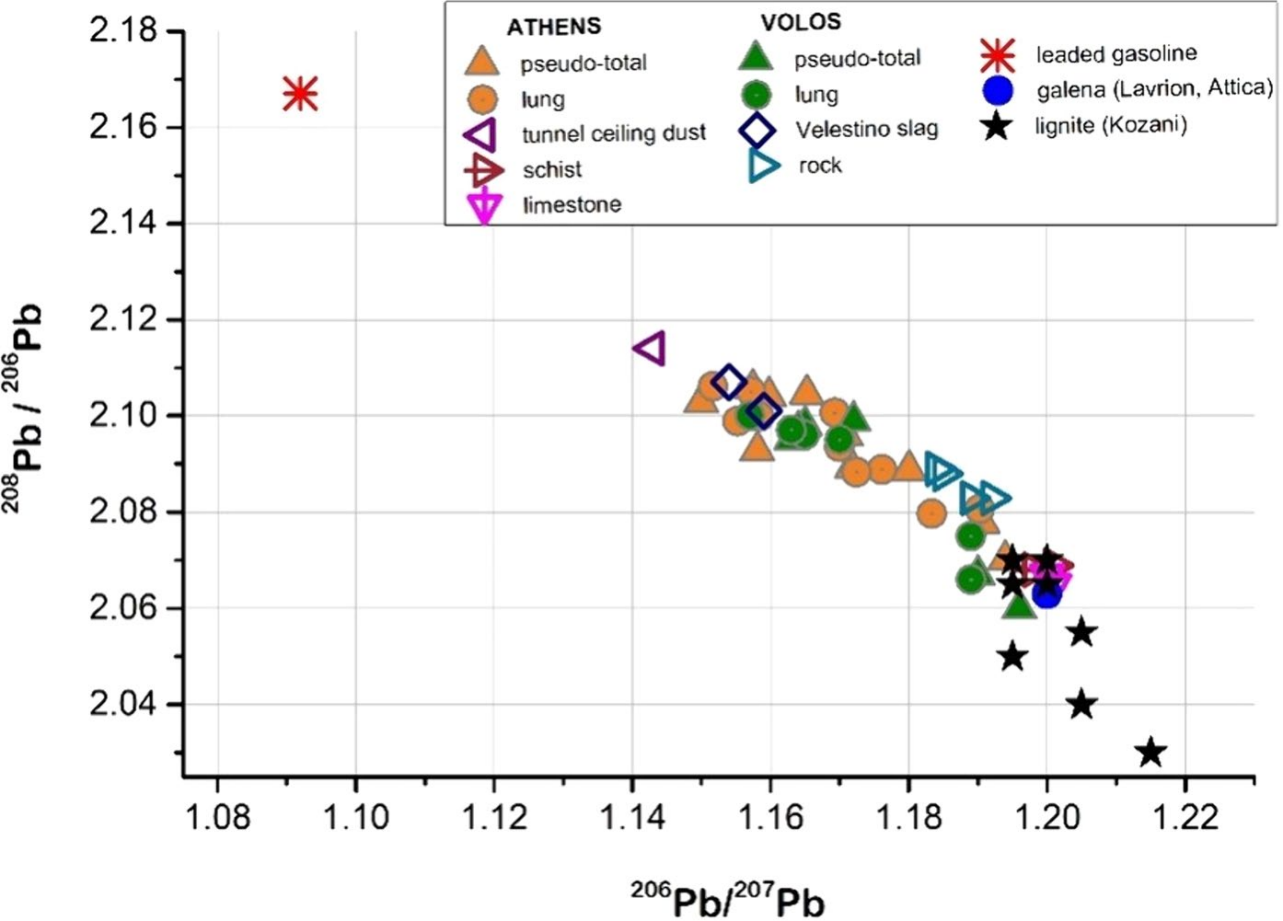
Pb isotope composition (^206^Pb / ^207^Pb vs ^208^Pb/.^206^Pb) for the pseudo-total and bioaccessible Pb in Athens and Volos soils, compared to the isotopic signatures of regional background and potential anthropogenic Pb sources (Kelepertzis et al., 2016, 2020). European leaded gasoline is a mean value from Erel et al. (1997), Komárek et al. (2008) Monna et al. (1999) and Teutsch et al. (2001). The Pb isotope composition of lignite samples from Kozani (North Greece) is also shown (Åberg et al., 2001)

The Pb isotope results for all the samples are plotted using the ^206^Pb/^207^Pb and ^208^Pb/^206^Pb ratios in Fig. 6, a valuable tool for tracing the sources of Pb, as outlined by Erel et al. (1997). Materials with distinctive Pb isotopic signatures from previous studies (Kelepertzis et al., 2016, 2020) were used as endmembers (Table S9 in Supplementary Material). Potential sources of Pb within the studied areas relate primarily to traffic-related Pb in the case of Athens, industrial activities for Volos area (steel emissions and coal use in cement production) and geogenic inputs from the weathering of regional bedrock. Although there is an overlap in the Pb isotope composition in the < 10 μm fraction between the soil in Athens and the soil in Volos, some differences exist related to the different contribution of the sources to the Pb content. Specifically, the Pb isotope ratios of both pseudo-total and bioaccessible Pb in soil from the highly urbanized region of Athens displayed a clear linear trend, extending between the isotopic composition of underlying bedrock and vehicular traffic emissions, as represented by the tunnel ceiling dust or the European leaded gasoline. This observation indicates that Pb in < 10 μm fraction is a result of mixture of Pb from parent materials and Pb from vehicular exhaust emissions, in accordance to previous studies appeared in the scientific literature (Giordano et al., 2024; Hiller et al., 2022). In fact, the composition field of European leaded gasoline was far from the Pb isotope composition of the < 10 μm soil fraction in Athens, demonstrating less impact of this Pb source on the pseudo-total and bioaccessible Pb. A striking feature is that few samples are plotted very close to the natural end member; considering the Pb elevated concentrations of these samples (Table S8 and Fig. 5), it may be inferred that they are principally influenced by nearby Pb mineralization (Stouraiti et al., 2019).

With regard to Volos soil, two cluster of samples are evident. The Pb isotope composition of the samples collected around the steel factory and within the urban center lay on a mixing line between the regional background and the industrial signal of steel emissions (Fig. 6). Specifically, this cluster of samples are plotted near the isotopic composition of slag points indicating that pseudo-total and bioaccessible Pb in < 10 μm fraction is dominated by emissions from the steel factory. The other cluster of samples, however, deviate from the mixing line between the Pb isotope composition of regional bedrock and the steel emissions signature. These samples, which have been collected around the cement factory, are plotted almost identically to the isotopic composition of lignite samples from Kozani (North Greece), demonstrating that Pb (both pseudo-total and lung bioaccessible) in the < 10 μm soil fraction is governed by coal combustion in the cement plant. For both areas, no systematic differences in the ^206^Pb/^207^Pb ratio between the inhalable fraction and the < 100 μm fraction was observed (Fig. S5 in Supplementary Material), suggesting that anthropogenic Pb is evenly distributed among the specific size fractions. This is in agreement with previous studies that reported the accumulation of anthropogenic Pb in both fine and larger particles (Bi et al., 2013; Jeong & Ra, 2021; Luo et al., 2022).

### Health risk assessment

The non-carcinogenic health risk was examined for all the studied elements. Arsenic, Cd, Cu, Pb, Mn, Ni and Zn can cause metal fume fever and pneumonitis at acute exposure. Also, As, Cd, Cr, Mn, Ni and Zn can cause Nasal septum perforation, Chronic Obstructive Lung Disease (COLD) and allergic asthma at airways (Nemery, 1990). Moreover, As, Cd, Cr, Pb and Ni were examined for carcinogenic risk because they can cause cancer. Additionally, all these metal(loid)s can cause extra-pulmonary toxicity (Kim et al., 2015; Nemery, 1990).

**Fig. 7:**
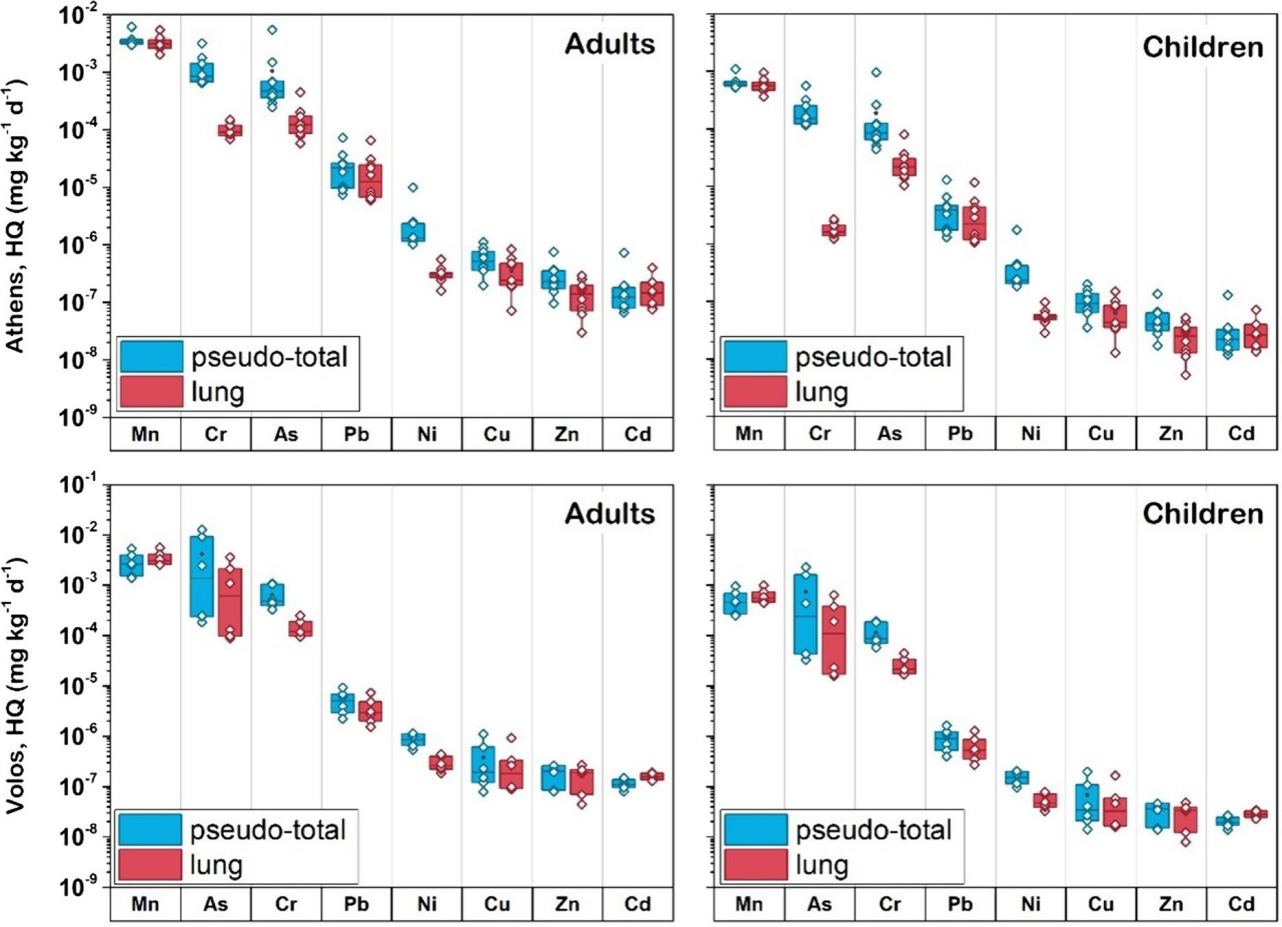
Results of HQ indices (non-carcinogenic risks) obtained for pseudo-total and lung metal(loid) concentrations in < 10 μm soil fraction for adults and children

Health risks were assessed for both pseudo-total and bioaccessible metal(loid) concentrations in < 10 μm soil fraction. The results of non-carcinogenic health risks are shown in Fig. 7. The values of HI were lower than 1 for both areas for adults and children, suggesting an acceptable non-carcinogenic risk due to metal(loid) exposure to the < 10 μm fraction. The HI values for adults were lower than those for children. The non-carcinogenic risk decreased in the order: Mn > Cr > As > Pb > Ni > Cu > Zn > Cd in Athens and Mn > As > Cr > Pb > Ni > Cu > Zn > Cd in Volos. Manganese was found to be the main contributor to the estimated health risks for both industrial and highly urbanized settings. The non-carcinogenic risks based on both pseudo-total and lung bioaccessible concentrations were quite similar for both areas. This observation indicates that anthropogenic metal(loid) sources related to traffic-related and industrial emissions do not have negative effects on human health when considering inhalation of < 10 μm soil particles.

**Fig. 8:**
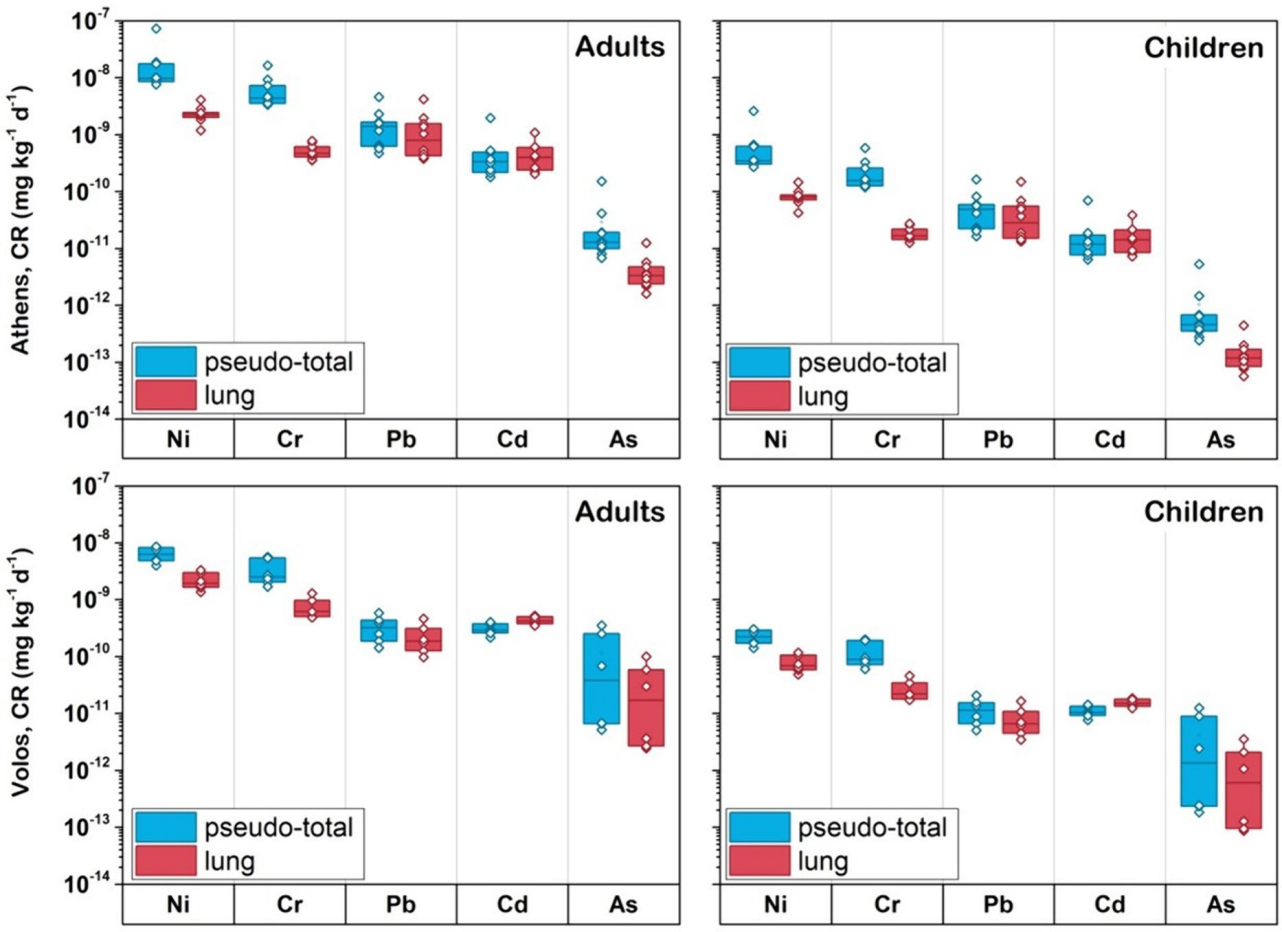
Results of cancer risk indexes obtained for pseudo-total and lung concentrations of considered carcinogenic metal(loids) in < 10 μm soil fraction for adults and children

In terms of carcinogenic risk, the TCR probabilities for As, Cd, Cr, Ni and Pb to adults and children were under the acceptable level (< 1 × 10^–4^) (Fig. 8), indicating no significant carcinogenic risks to adults and children exposed to < 10 μm in soil. It is noted that Cu, Mn and Zn are non-carcinogenic and thus no data is shown for these elements. Nickel was the dominant contributor to the cumulative carcinogenic risk, followed, mostly by Cr for both areas (Fig. S6 in Supplementary Material). The low non-carcinogenic and carcinogenic risks determined via the inhalation pathway in the present study are in accordance with similar results published in the literature (Luo et al., 2022; Yan et al., 2021).

## Conclusions

In this comprehensive study, the inhalable (< 10 μm) fraction of contaminated soil from highly urbanized and industrial environments was characterized in terms of its magnetic signature, pseudo-total and lung bioaccessible metal(loid) content and corresponding Pb isotope analyses, and human health risk assessment. Magnetic analysis revealed that the dominant magnetic carrier is impure magnetite. Cadmium and Mn were the elements that showed the highest bioaccessibility for both settings. The Pb isotope analyses demonstrated that anthropogenic Pb in the < 10 μm soil fraction is related to vehicular sources for the highly urbanized environment. For the industrial setting, industrial emissions from the steel factory and coal combustion in cement plant were dominant contributors to Pb accumulation in < 10 μm soil fraction. There were no differences in the Pb isotope ratios between pseudo-total and lung bioaccessible Pb, highlighting that anthropogenic Pb fingerprint is well-delineated in simulated lung solutions. The performed inhalation risk assessment suggested that the non-carcinogenic and carcinogenic human health risks of metal(loids) in both areas were below those considered a risk. Manganese was the major contributor to non-carcinogenic risks, and Ni was the dominant contributor to carcinogenic risk. These findings contribute valuable insights into the complex dynamics in urban and industrial soils, emphasizing the importance of a thorough characterization of the inhalable fraction when considering exposure of humans to metal(loids).

### Supplementary Information

Below is the link to the electronic supplementary material.

Supplementary file1 (DOCX 1174 KB)
