# Supplementary material for: Characterization of the inhalable fraction (< 10 μm) of soil from highly urbanized and industrial environments: magnetic measurements, bioaccessibility, Pb isotopes and health risk assessment
Source: Environ Geochem Health. 2024 Jun 7;46(7):230. doi: 10.1007/s10653-024-02009-z (PMC11161548; doi:10.1007/s10653-024-02009-z)
Supplement: Supplementary file 1 — Supplementary file1 (DOCX 1174 KB) [file 10653_2024_2009_MOESM1_ESM.docx]

**Characterization of the inhalable fraction (<10 μm) of soil from highly urbanized and industrial environments: magnetic measurements, bioaccessibility, Pb isotopes and health risk assessment**

Stavroula Menegaki^1^, Efstratios Kelepertzis^1^*, Zacharenia Kypritidou^1^, Anastasia Lampropoulou^1^, Vladislav Chrastný^2^, Elina Aidona^3^, Anna Bourliva^4^, Michael Komárek^2^

^1^Department of Geology and Geoenvironment, National and Kapodistrian University of Athens, Panepistimiopolis, Zographou, 15784, Athens, Greece

^2^Department of Environmental Geosciences, Faculty of Environmental Sciences, Czech University of Life Sciences Prague, Kamýcká 129, 165 00, Prague-Suchdol, Czech Republic

^3^Department of Geophysics, Faculty of Geology, School of Geology, Aristotle University of Thessaloniki, Thessaloniki, Greece

^4^Directorate of Secondary Education of Western Thessaloniki, 56430 Thessaloniki, Greece;

* corresponding author: Efstratios Kelepertzis ([kelepert@geol.uoa.gr](mailto:kelepert@geol.uoa.gr)), +30 2107274867

**Supplementary Material**

**Figure S1:** Representative Scanning Electron Microscope (SEM) photomicrographs in back-scattered mode of selected soil samples after the extraction method used to separate the inhalable fraction (<10 μm).












**Figure S2:** Representative thermomagnetic curves of selected soil samples in <10 μm fraction.

**
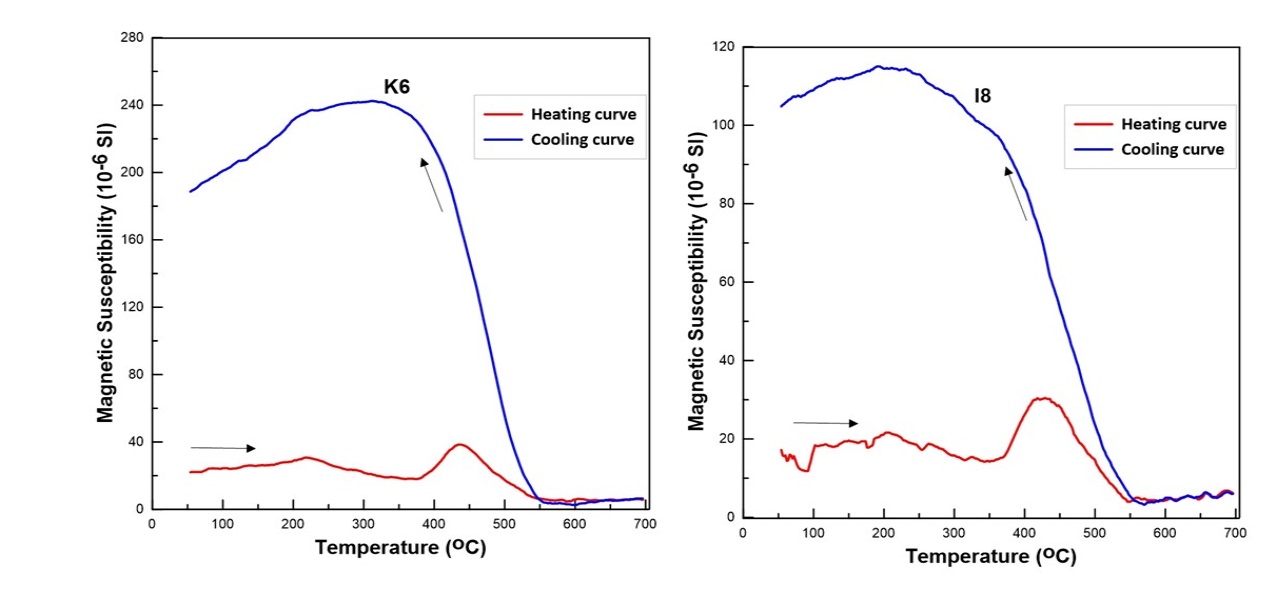
**

**Figure S3:** Variations in the pseudo-total concentration of metal(loid)s in Athens and Volos soil between <10 μm and <100 μm size fractions. Data for <100 μm fraction are from Kelepertzis and Argyraki (2015) for Athens soil and Kelepertzis et al (2020) for Volos soil.


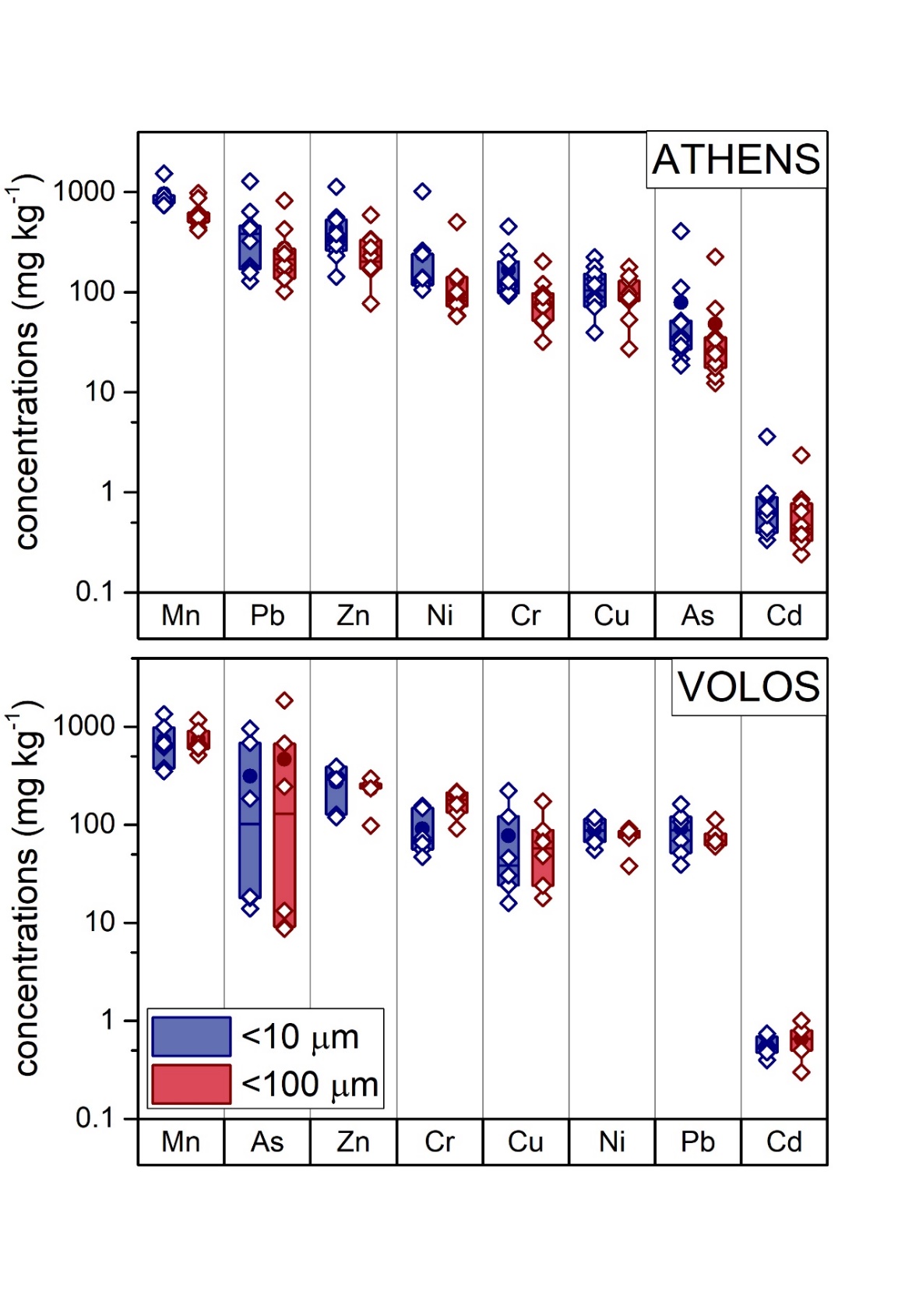


**Figure S4:** Variations in the lung bioaccessibility (both as concentrations and as percentage of the pseudo-total content) of metal(loid)s in Volos soil between <10 μm and <100 μm size fraction. Data for <100 μm are from Kelepertzis et al. (2021).


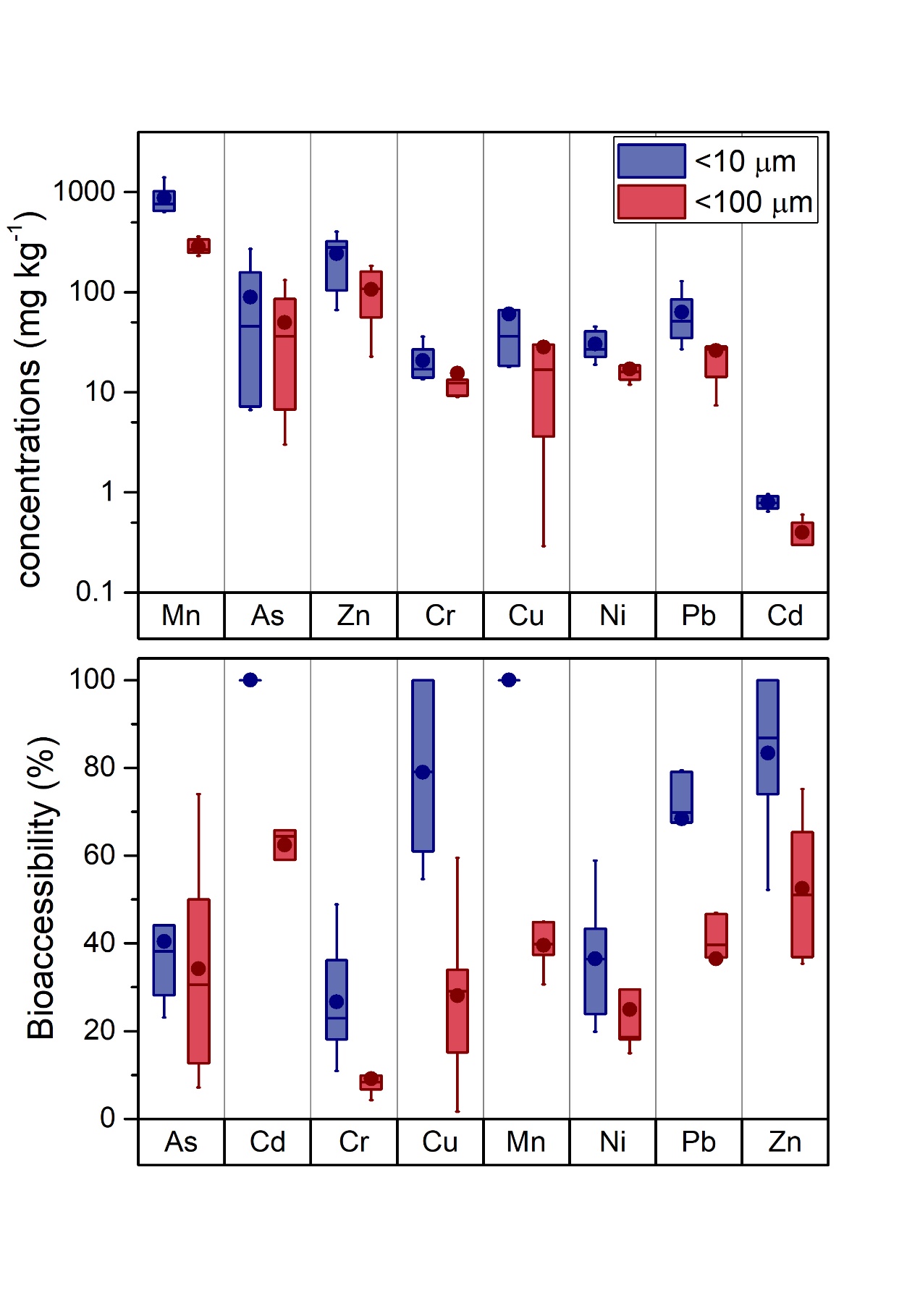


**Figure S5:** Comparison between ^206^Pb/^207^Pb ratios for pseudo-total Pb in <10 μm (present study) and total Pb in <100 μm (data are from Kelepertzis et al. (2016) and Kelepertzis et al. (2020).

**
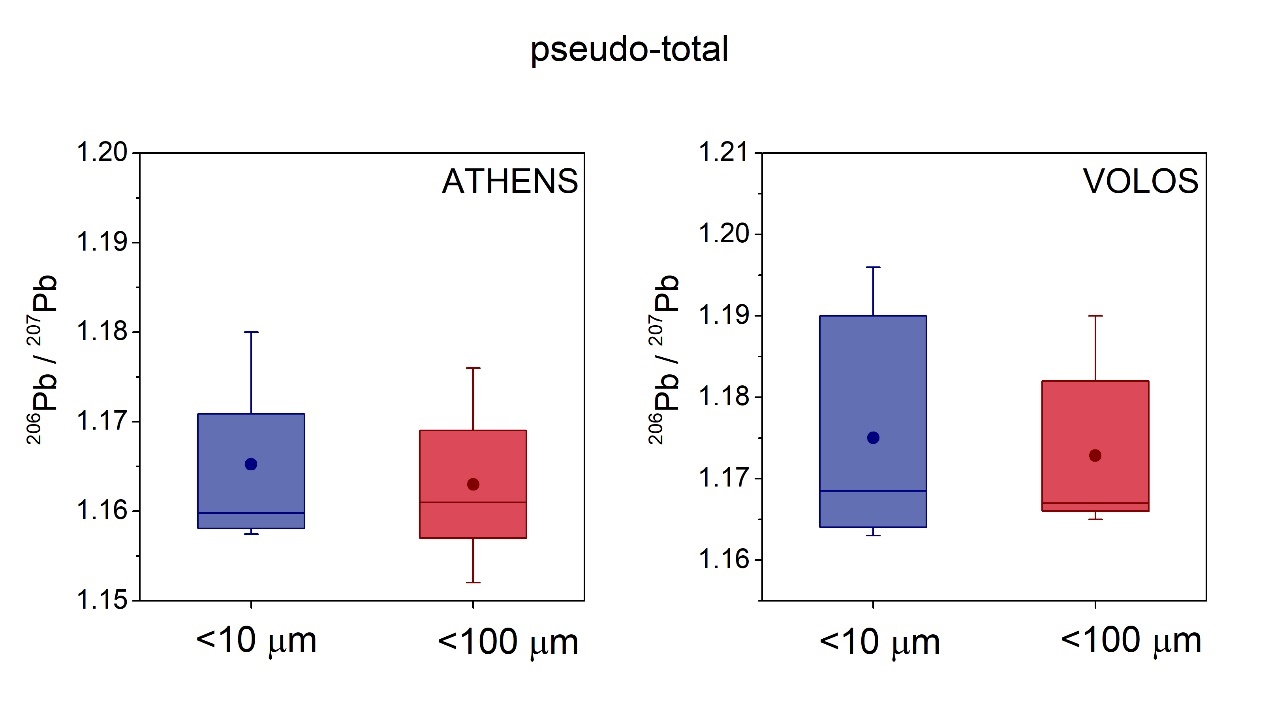
**

**Figure S6:** Contribution of each element to total carcinogenic risk (TCR) based on pseudo-total and lung concentrations of metal(loids), for adults and children.


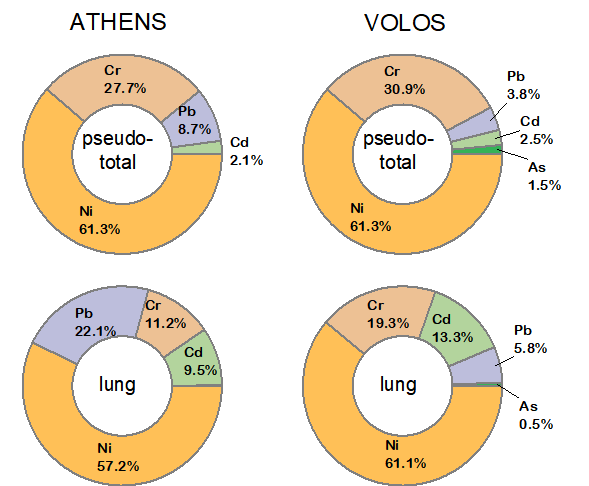


**Table S1:** Quality control results for the EPA 3050B and ALF methods based on analytical duplicates (the letters A and B for each sample code denote the analytical duplicates). Concentrations are in mg kg^-1^.

| **EPA 3050B** | | | | | | | | |
| --- | --- | --- | --- | --- | --- | --- | --- | --- |
| **sample** | **As** | **Cd** | **Cr** | **Cu** | **Mn** | **Ni** | **Pb** | **Zn** |
| F14A | 22 | 0,3 | 92 | 89 | 735 | 130 | 129 | 265 |
| F14B | 21 | 0,3 | 93 | 89 | 743 | 129 | 129 | 259 |
|  |  |  |  |  |  |  |  |  |
| I15A | 33 | 0,4 | 128 | 132 | 825 | 137 | 182 | 314 |
| I15B | 34 | 0,4 | 133 | 138 | 860 | 143 | 183 | 328 |
|  |  |  |  |  |  |  |  |  |
| L20A | 33 | 1 | 147 | 80 | 836 | 155 | 175 | 425 |
| L20B | 26 | 1 | 109 | 62 | 650 | 122 | 141 | 340 |
|  |  |  |  |  |  |  |  |  |
| V45A | 14 | 1 | 56 | 24 | 619 | 55 | 108 | 318 |
| V45B | 7 | 0,31 | 26 | 12 | 288 | 26 | 51 | 158 |
|  |  |  |  |  |  |  |  |  |
| V33A | 147 | 0,32 | 51 | 38 | 304 | 48 | 45 | 192 |
| V33B | 223 | 0,47 | 74 | 54 | 453 | 70 | 69 | 290 |

| **ALF** | | | | | | | | |
| --- | --- | --- | --- | --- | --- | --- | --- | --- |
| **Sample** | **As** | **Cd** | **Cr** | **Cu** | **Mn** | **Ni** | **Pb** | **Zn** |
| F14A | 6 | 0,4 | 10 | 43 | 650 | 17 | 106 | 130 |
| F14B | 6 | 0,4 | 10 | 43 | 656 | 16 | 103 | 119 |
|  |  |  |  |  |  |  |  |  |
| L20A | 13 | 1 | 21 | 48 | 751 | 34 | 114 | 298 |
| L20B | 13 | 1 | 22 | 49 | 753 | 34 | 112 | 293 |
|  |  |  |  |  |  |  |  |  |
| J10A | 17 | 0,4 | 11 | 41 | 951 | 35 | 131 | 118 |
| J10B | 14 | 0,3 | 9 | 34 | 797 | 29 | 105 | 98 |
|  |  |  |  |  |  |  |  |  |
| J7A | 4 | 0,5 | 12 | 41 | 531 | 40 | 1181 | 44 |
| J7B | 4 | 0,5 | 11 | 38 | 494 | 38 | 1144 | 45 |
|  |  |  |  |  |  |  |  |  |
| K6A | 9 | 2 | 20 | 17 | 1149 | 66 | 147 | 107 |
| K6B | 7 | 2 | 14 | 12 | 863 | 47 | 108 | 80 |
|  |  |  |  |  |  |  |  |  |
| V45A | 10 | 1 | 15 | 18 | 649 | 15 | 71 | 268 |
| V45B | 10 | 1 | 14 | 21 | 624 | 22 | 24 | 280 |
|  |  |  |  |  |  |  |  |  |
| V33A | 80 | 1 | 34 | 52 | 687 | 42 | 54 | 315 |
| V33B | 84 | 1 | 37 | 55 | 712 | 40 | 56 | 335 |
|  |  |  |  |  |  |  |  |  |
| V37A | 261 | 1 | 16 | 20 | 621 | 28 | 26 | 99 |
| V37B | 279 | 1 | 17 | 20 | 646 | 30 | 28 | 109 |
|  |  |  |  |  |  |  |  |  |

**Table S2:** Quality control results for the pseudo-total content of metal(loids) based on certified reference material (SRM 2709a). Concentrations in mg kg^-1^.

| Element | Measured (average n=3) | Certified | Recovery (%) |
| --- | --- | --- | --- |
| As | 8.8 | 10.5 ± 0.3 | 84 |
| Cd | 0.315 | 0.371 ± 0.002 | 85 |
| Cr | 72 | 130 ± 9 | 55 |
| Cu | 30.8 | 33.9 ± 0.5 | 91 |
| Mn | 452 | 529 ± 18 | 85 |
| Ni | 74 | 85 ± 2 | 87 |
| Pb | 14.3 | 17.3 ± 0.1 | 83 |
| Zn | 97.7 | 103 ± 4 | 95 |

**Table S3:** Chemical composition of Artificial lysosomal fluid used for in vitro bioaccessibility experiments (Guney et al., 2016).

| **Chemical constituent** | **Amount (g/L)** |
| --- | --- |
| Magnesium chloride (MgCl_2_) | 0.0503 |
| Sodium chloride (NaCl) | 3.2106 |
| Calcium chloride (CaCl_2_) | 0.1282 |
| Sodium sulfate (Na_2_SO_4_) | 0.0391 |
| Disodium phosphate (Na_2_HPO_4_∙7H_2_O) | 0.1342 |
| Sodium citrate dihydrate (C_6_H_5_Na_3_O_7_∙2H_2_O) | 0.0906 |
| Sodium hydroxide (NaOH) | 6.0024 |
| Citric acid (C_6_H_8_O_7_) | 20.801 |
| Glycine (NH_2_CH_2_COOH) | 0.0593 |
| Sodium tartrate dihydrate (C_6_H_4_KNaO_6_∙4H_2_O) | 0.1105 |
| Sodium lactate (C_3_H_5_NaO) | 0.0852 |
| Sodium pyruvate (C_3_H_3_Ο_3_Na) | 0.0863 |

**Table S4:** Definition and reference values of parameters for non-carcinogenic and carcinogenic health risk assessment of metal(loids) in the <10 μm soil fraction.

| **Parameters** | **Definition** | **Units** | **Values** |  | **Reference** |
| --- | --- | --- | --- | --- | --- |
|  |  |  | **Adult** | **Child** |  |
| **InhR** | Soil inhalation rate | m^3^/day | 20 | 7.6 | Van den Berg (1995);  Zheng et al. (2010) |
| **EF** | Exposure frequency | day/year | 350 | 350 | USEPA (2002) |
| **ED** | Exposure duration | year | 30 | 6 | USEPA (2002) |
| **BW** | Body weight | kg | 70 | 15 | USEPA (2002) |
| **AT** | Average time | day | 365×ED (noncarcinogen) // 365×70 (carcinogen) | | USEPA (2002) |
| **PEF** | Soil to air particulate emission factor | m^3^/kg | 1.36 x 10^9^ | 1.36 x 10^9^ | USEPA (2002) |

**Table S5:** Summary of references dose (RfD) and slope factor (IUR) for the non-carcinogenic and carcinogenic risk assessment of metal(oid)s.

| **Metal(loids)** | **RfDinh (mg kg^-1^ day^-1^)** | **IUR (mg kg^-1^ day^-1^)** |
| --- | --- | --- |
| **As** | 1.5x10^-5^ | 4.3x10^-3^ |
| **Cd** | 1x10^-3^ | 6.3 |
| **Cr** | 2.86x10^-5^ | 4.2x10^1^ |
| **Cu** | 4.02x10^-2^ |  |
| **Mn** | 5x10^-5^ |  |
| **Ni** | 2.06x10^-2^ | 8.4x10^-1^ |
| **Pb** | 3.52x10^-3^ | 4.2x10^-2^ |
| **Zn** | 3x10^-1^ |  |
| **References** | USEPA (1993, 2010) | USEPA 2001a, 2001b |
|  | As from Huang et al. (2014) | |

**Table S6:** Comparison of pseudo-total concentrations in < 10 μm soil fraction in the present study with similar data published in the literature.

| **Pseudo-total (mg kg^-1^)** | | | | | | | | | | | |
| --- | --- | --- | --- | --- | --- | --- | --- | --- | --- | --- | --- |
| **Reference Area** | **Area Type** | **As** | **Cd** | **Cr** | **Cu** | **Mn** | **Ni** | **Pb** | **Zn** | **Size** | **Reference** |
| Torino, Italy | Urban | 18 | 1.3 | 358 | 147 | - | 408 | 286 | 396 | <10μm | (Yan et al. 2021) |
| France and Canada | Contaminated | 34.75 | - | - | 45.6 | 1325 | 105.1 | 107.6 | 364.5 | <20μm | (Guney et al. 2017) |
| Piracicaba, Santo Amaro, Apiaí, Brazil | Industrial- Urban | - | 4.6 | - | 100 | 600 | - | 400 | 600 | <10μm | (Boim et al. 2021) |
| Sevila, Spain | Urban | - | - | 399 | 493 | - | 350 | 1410 | 770 | 2-10μm | (Madrid et al. 2008) |
| Torino, Italy | Urban | - | - | 36 | 219 | - | 32 | 599 | 466 | 2-10μm | (Madrid et al. 2008) |
| European topsoils mean |  | 11.6 | 0.28 | 98.4 | 17.3 | 524 | 37 | 32 | 68.1 |  | FOREGS 2005 |
| Worldwide soils |  | 6.83 | 0.41 | 59.5 | 38.9 | 488 | 29 | 27 | 70 |  | (Kabata-Pendias 2011) |
| Athens, Greece | Urban | 35 | 0.61 | 121 | 105 | 835 | 134 | 382 | 341 | <10μm | this study |
| Volos, Greece | Industrial | 102 | 0.54 | 69 | 38 | 645 | 87 | 88 | 304 | <10μm | this study |

**Table S7:** Comparison of lung bioaccessible concentrations in < 10 μm soil fraction in the present study with similar data published in the literature.

| **Lung bioaccesibility (mg kg^-1^)** | | | | | | | | | | | | |
| --- | --- | --- | --- | --- | --- | --- | --- | --- | --- | --- | --- | --- |
| **Reference Area** | **Area Type** | **As** | **Cd** | **Cr** | **Cu** | **Mn** | **Ni** | **Pb** | **Zn** | **Solution** | **Size** | **Reference** |
| Torino, Italy | Urban | 2 | 0.6 | 5.2 | 47.9 | - | 38.8 | 123.7 | 65.5 | Gamble | <10μm | (Yan et al. 2021) |
| France and Canada | Contaminated | 1.76 | - | - | 3.47 | 48.25 | 11.2 | 7.54 | 18.85 | ALF | <20μm | (Guney et al. 2017) |
| Piracicaba, Santo Amaro, Apiaí, Brazil | Industrial- Urban | - | 4.2 | - | 100 | 400 | - | 400 | 500 | ALF | <10μm | (Boim et al. 2021) |
| Athens, Greece | Urban | 9 | 0.73 | 13 | 48 | 777 | 31 | 218 | 208 | ALF | <10μm | this study |
| Volos, Greece | Industrial | 46 | 0.78 | 17 | 37 | 764 | 27 | 52 | 282 | ALF | <10μm | this study |

**Table S8:** Pb concentrations and isotope compositions of pseudo-total and bioaccessible analyses in <10 μm soil fraction from the studied areas.

| **Pseudo-total, Athens <10 μm** | | | |
| --- | --- | --- | --- |
| **Sample** | **Pb concentration** | **^206^Pb / ^207^Pb** | **^208^Pb / ^206^Pb** |
| J10 | 172 | 1.180 | 2.089 |
| K10 | 458 | 1.157 | 2.106 |
| L20 | 158 | 1.171 | 2.096 |
| J11 | 462 | 1.158 | 2.093 |
| H12 | 325 | 1.160 | 2.104 |
| K6 | 438 | 1.194 | 2.070 |
| F14 | 129 | 1.172 | 2.089 |
| I8 | 638 | 1.150 | 2.103 |
| J7 | 1282 | 1.165 | 2.105 |
| I15 | 183 | 1.191 | 2.078 |
| **ALF, Athens <10 μm** | | | |
| **Sample** | **Pb concentration** | **^206^Pb / ^207^Pb** | **^208^Pb / ^206^Pb** |
| J10 | 118 | 1.176 | 2.089 |
| K10 | 383 | 1.157 | 2.105 |
| L20 | 113 | 1.170 | 2.094 |
| J11 | 428 | 1.155 | 2.099 |
| H12 | 289 | 1.158 | 2.100 |
| K6 | 128 | 1.190 | 2.081 |
| F14 | 104 | 1.172 | 2.088 |
| I8 | 541 | 1.152 | 2.106 |
| J7 | 1163 | 1.169 | 2.101 |
| I15 | 147 | 1.183 | 2.080 |
| **Pseudo-total, Volos <10 μm** | | | |
| **Sample** | **Pb concentration** | **^206^Pb / ^207^Pb** | **^208^Pb / ^206^Pb** |
| V18 | 163 | 1.165 | 2.098 |
| V29 | 121 | 1.164 | 2.097 |
| V33 | 69 | 1.163 | 2.095 |
| V35 | 52 | 1.190 | 2.067 |
| V37 | 39 | 1.196 | 2.062 |
| V45 | 108 | 1.172 | 2.099 |
| **ALF, Volos <10 μm** | | | |
| **Sample** | **Pb concentration** | **^206^Pb / ^207^Pb** | **^208^Pb / ^206^Pb** |
| V18 | 129 | 1.165 | 2.096 |
| V29 | 85 | 1.163 | 2.097 |
| V33 | 55 | 1.157 | 2.100 |
| V35 | 35 | 1.189 | 2.075 |
| V37 | 27 | 1.189 | 2.066 |
| V45 | 48 | 1.170 | 2.095 |

**Table S9:** Pb isotope compositions of regional background and potential anthropogenic Pb sources, published by Kelepertzis et al. (2016) and Kelepertzis et al. (2020). European leaded gasoline is a mean value from Erel et al. (1997), Komárek et al. (2008) Monna et al. (1999) and Teutsch et al. (2001). The Pb isotope compositions of lignite are from Åberg et al. (2001).

| **Sample** | **^206^Pb / ^207^Pb** | **^208^Pb / ^206^Pb** |
| --- | --- | --- |
| Galena (Lavrio) | 1.200 | 2.063 |
| Schist (Athens) | 1.198 | 2.068 |
| Schist (Athens) | 1.201 | 2.069 |
| Limestone (Athens) | 1.200 | 2.067 |
| Limestone (Athens) | 1.201 | 2.065 |
| Velestino slag | 1.154 | 2.107 |
| Velestino slag 2 | 1.159 | 2.101 |
| Rock (Volos) | 1.185 | 2.088 |
| Rock (Volos) | 1.192 | 2,083 |
| Rock (Volos) | 1.184 | 2.089 |
| Rock (Volos) | 1.189 | 2,083 |
| Leaded gasoline | 1.092 | 2.167 |
| Tunnel ceiling dust (Athens) | 1.143 | 2.114 |
| Lignite, Kozani | 1.200 | 2.070 |
| Lignite, Kozani | 1.195 | 2.070 |
| Lignite, Kozani | 1.195 | 2.065 |
| Lignite, Kozani | 1.200 | 2.065 |
| Lignite, Kozani | 1.205 | 2.055 |
| Lignite, Kozani | 1.195 | 2.050 |
| Lignite, Kozani | 1.205 | 2.040 |
| Lignite, Kozani | 1.215 | 2.030 |

**References:**

Åberg, G., Charalambides, G., Fosse, G., Hjelmseth, H., 2001. The use of Pb isotopes to differentiate between contemporary and ancient sources of pollution in Greece. *Atmospheric Environment*, *35*, 4609-4615.

Boim, A. G. F., Patinha, C., Wragg, J., Cave, M., & Alleoni, L. R. F. (2021). Respiratory bioaccessibility and solid phase partitioning of potentially harmful elements in urban environmental matrices. *Science of The Total Environment*, *765*, 142791. https://doi.org/10.1016/j.scitotenv.2020.142791

Erel, Y., Veron, A., Halicz, L., 1997. Tracing the Transport of Anthropogenic Lead in the Atmosphere and in Soils Using Isotopic Ratios. *Geochimica et Cosmochimica Acta,* *61*, 4495–4505. <https://doi.org/10.1016/S0016-7037(97)00353-0>.

FOREGS. (2005). Forum of the European Geological Survey Directors. Geochemical Atlas of Europe, Geological Survey of Finland, Espoo.

Guney, M., Bourges, C. M.-J., Chapuis, R. P., & Zagury, G. J. (2017). Lung bioaccessibility of As, Cu, Fe, Mn, Ni, Pb, and Zn in fine fraction (< 20 μm) from contaminated soils and mine tailings. *Science of The Total Environment*, *579*, 378–386. https://doi.org/10.1016/j.scitotenv.2016.11.086

Guney, M., Chapuis, R.P., Zagury, G.J., 2016. Lung Bioaccessibility of Contaminants in Particulate Matter of Geological Origin. *Environmental Science and Pollution Research,* *23*, 24422–24434. <https://doi.org/10.1007/s11356-016-6623-3>.

Hernández-Pellón, A., Nischkauer, W., Limbeck, A., & Fernández-Olmo, I. (2018). Metal(loid) bioaccessibility and inhalation risk assessment: A comparison between an urban and an industrial area. *Environmental Research*, *165*, 140–149. https://doi.org/10.1016/j.envres.2018.04.014

Huang, M., Wang, W., Chan, C. Y., Cheung, K. C., Man, Y. B., Wang, X., & Wong, M. H. (2014). Contamination and risk assessment (based on bioaccessibility via ingestion and inhalation) of metal(loid)s in outdoor and indoor particles from urban centers of Guangzhou, China. Science of The Total Environment, 479–480, 117–124. <https://doi.org/10.1016/j.scitotenv.2014.01.115>

Kabata-Pendias, A. (2011). *Trace elements in soils and plants* (4th ed.). Boca Raton: CRC Press.

Kelepertzis, E., Argyraki., 2015. Geochemical Associations for Evaluating the Availability of Potentially Harmful Elements in Urban Soils: Lessons Learnt from Athens, Greece. *Applied Geochemistry,* *59*, 63–73. <https://doi.org/10.1016/j.apgeochem.2015.03.019>.

Kelepertzis, E., Komárek, M., Argyraki, A., Šillerová, H., 2016. Metal(Loid) Distribution and Pb Isotopic Signatures in the Urban Environment of Athens, Greece. *Environmental Pollution,* *213*, 420–31. <https://doi.org/10.1016/j.envpol.2016.02.049>.

Kelepertzis, E., Argyraki, A., Chrastný, V., Botsou, F., Skordas, K., Komárek, M., Fouskas, A., 2020. Metal(Loid) and Isotopic Tracing of Pb in Soils, Road and House Dusts from the Industrial Area of Volos (Central Greece). *Science of The Total Environment,* *725*: 138300. <https://doi.org/10.1016/j.scitotenv.2020.138300>.

Kelepertzis, E., Chrastný, V., Botsou, F., Sigala, E., Kypritidou, Z., Komárek, M., Skordas, K., Argyraki, A., 2021. Tracing the Sources of Bioaccessible Metal(Loid)s in Urban Environments: A Multidisciplinary Approach. *Science of The Total Environment,* *771*: 144827.

Komárek, M,, Ettler, V., Chrastný, V., Mihaljevič, M., 2008. Lead Isotopes in Environmental Sciences: A Review. *Environment International,* *34*, 562–77. <https://doi.org/10.1016/j.envint.2007.10.005>.

Madrid, F., Biasioli, M., & Ajmone-Marsan, F. (2008). Availability and Bioaccessibility of Metals in Fine Particles of Some Urban Soils. *Archives of Environmental Contamination and Toxicology*, *55*(1), 21–32. https://doi.org/10.1007/s00244-007-9086-1

Monna, F, Aiuppa, A., Varrica, D., Dongarra, G., 1999. Pb Isotope Composition in Lichens and Aerosols from Eastern Sicily: Insights into the Regional Impact of Volcanoes on the Environment. *Environmental Science & Technology,* *33*, 2517–2523. <https://doi.org/10.1021/es9812251>.

Teutsch, N., Erel, Y., Halicz, L., Banin, A., 2001. Distribution of Natural and Anthropogenic Lead in Mediterranean Soils. *Geochimica et Cosmochimica Acta,* *65*, 2853–64. <https://doi.org/10.1016/S0016-7037(01)00607-X>

USEPA, 1993. Reference dose (RfD): description and use in health risk assessments. Background Document 1 A. Integrated risk information system(IRIS).

USEPA, 2001a. Child-Specific Exposure Factors Handbook. National Center for Environmental Assessment. EPA-600-P-00-002B.

USEPA, 2001b. Risk Assessment Guidance for Superfund: Volume III-Part A, Process for Conducting Probabilistic Risk Assessment. Washington, D.C. EPA 540-R-02-002.

USEPA, 2002. Supplemental Guidance for Developing Soil Screening Levels for Superfund Sites. U.S. Environmental Protection Agency, Office of Emergency and Remedial Response, Washington.

USEPA, 2010. Estimation of Relative Bioavailablity of Lead in Soil and Soil-like Materials Using in Vivo and in Vitro Methods. OSWER 9285.7-77. Office of Solid Waste and Emergency Response, U.S. Environmental Protection Agency, Washington, DC.

Van den Berg, R., 1995. Human Exposure to Soil Contamination: a Qualitative and Quantitative Analysis towards Proposals for Human Toxicological Intervention Values. RIVM Report no. 725201011. Bilthoven, the Netherlands: National Institute of Public Health and Environmental Protection (RIVM).

Yan, L., Franco, A.-M., & Elio, P. (2021). Health risk assessment via ingestion and inhalation of soil PTE of an urban area. *Chemosphere*, *281*, 130964. https://doi.org/10.1016/j.chemosphere.2021.130964

Zheng, N., Liu, J., Wang, Q., & Liang, Z. (2010). Health risk assessment of heavy metal exposure to street dust in the zinc smelting district, Northeast of China. Science of the Total Environment, 408, 726–733.
